# Structure-Based Design of Small Imine Reductase Panels for Target Substrates

**DOI:** 10.1021/acscatal.3c02278

**Published:** 2023-09-05

**Authors:** Yuqi Yu, Arnau Rué Casamajo, William Finnigan, Christian Schnepel, Rhys Barker, Charlotte Morrill, Rachel S. Heath, Leonardo De Maria, Nicholas J. Turner, Nigel S. Scrutton

**Affiliations:** † Department of Chemistry, The University of Manchester, Manchester Institute of Biotechnology, 131 Princess Street, Manchester M1 7DN, U.K.; ‡ Augmented Biologics Discovery & Design, Department of Biologics Engineering, BioPharmaceuticals R&D, AstraZeneca, Cambridge CB21 6GH, U.K.; § Medicinal Chemistry, Research and Early Development, Respiratory and Immunology (RI), BioPharmaceuticals R&D, AstraZeneca, Gothenburg 43150, Sweden

**Keywords:** biocatalysis, enzyme screening, imine reductase, computational workflow, structural modeling

## Abstract

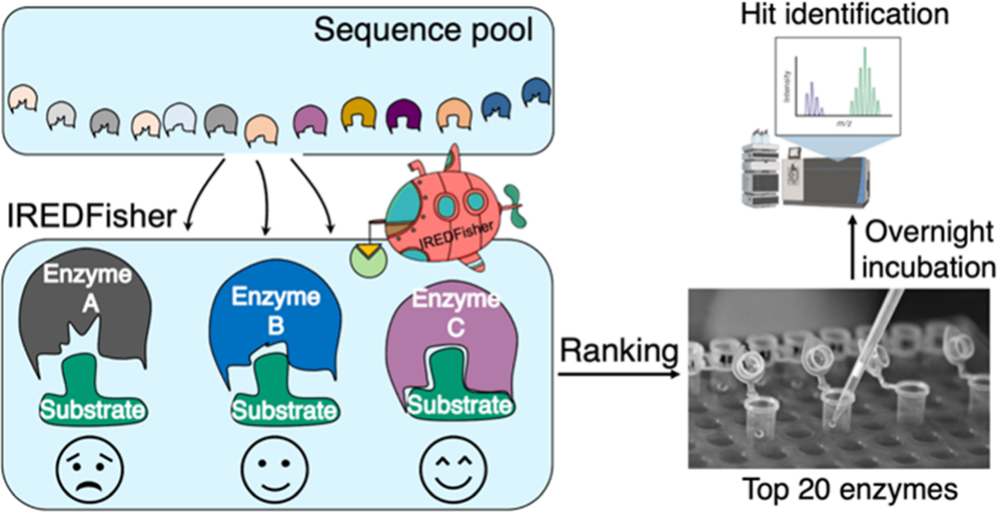

Biocatalysis is important in the discovery, development, and manufacture of pharmaceuticals. However, the identification of enzymes for target transformations of interest requires major screening efforts. Here, we report a structure-based computational workflow to prioritize protein sequences by a score based on predicted activities on substrates, thereby reducing a resource-intensive laboratory-based biocatalyst screening. We selected imine reductases (IREDs) as a class of biocatalysts to illustrate the application of the computational workflow termed IREDFisher. Validation by using published data showed that IREDFisher can retrieve the best enzymes and increase the hit rate by identifying the top 20 ranked sequences. The power of IREDFisher is confirmed by computationally screening 1400 sequences for chosen reductive amination reactions with different levels of complexity. Highly active IREDs were identified by only testing 20 samples in vitro. Our speed test shows that it only takes 90 min to rank 85 sequences from user input and 30 min for the established IREDFisher database containing 591 IRED sequences. IREDFisher is available as a user-friendly web interface (https://enzymeevolver.com/IREDFisher). IREDFisher enables the rapid discovery of IREDs for applications in synthesis and directed evolution studies, with minimal time and resource expenditure. Future use of the workflow with other enzyme families could be implemented following the modification of the workflow scoring function.

## Introduction

**Figure 1:**
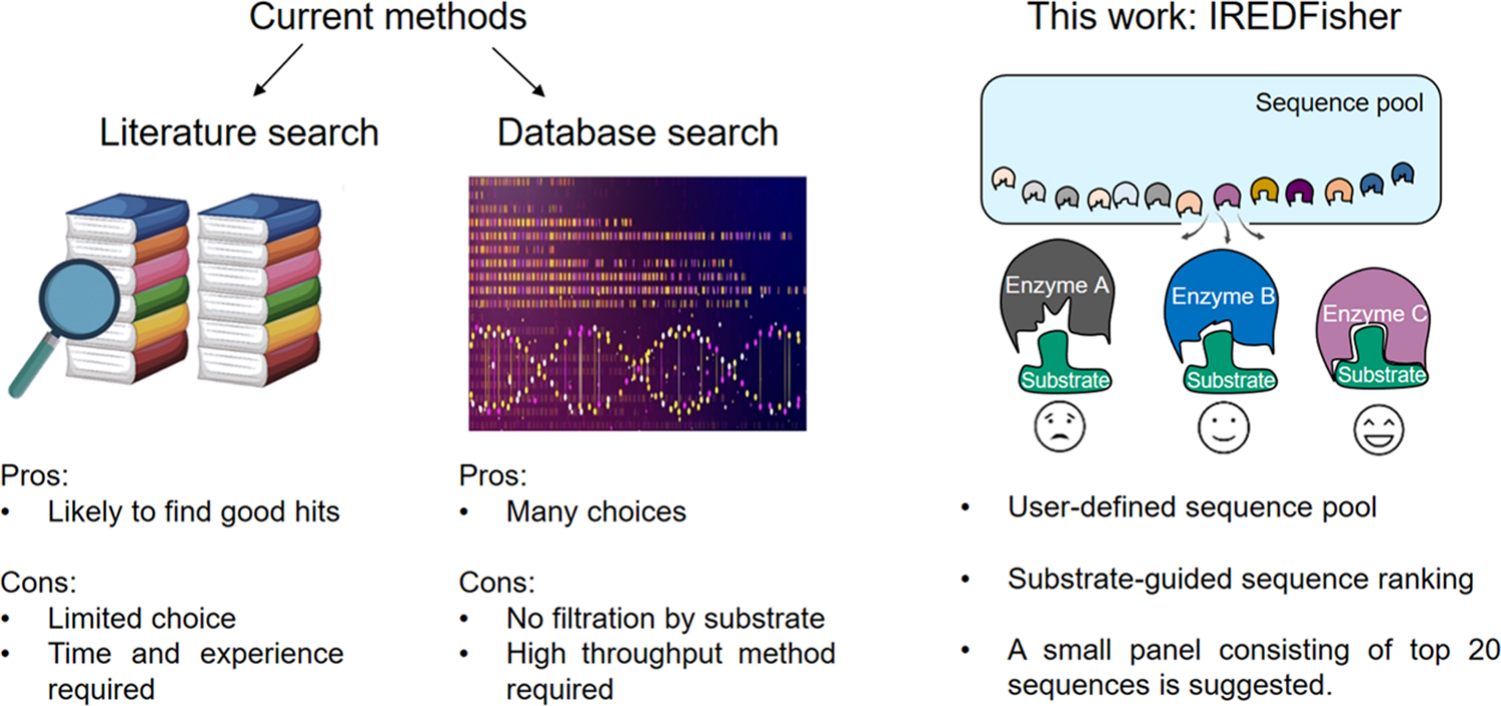
Solutions of enzyme discovery for targeted substrate(s). Left: two common methods for finding hit enzymes for targeted substrate(s). Right: a schematic diagram of IREDFisher developed in this work. Input sequences are filtered by IREDFisher with the aid of structural modeling and molecular docking, which allows the user to obtain a small panel consisting of prioritized sequences.

Enzymatic synthesis has gained growing attention in recent years due to the inherent advantages of benign reaction conditions along with exquisite chemo-, stereo-, and regio-selectivity.^1−5^ However, finding the optimal biocatalyst to effect the transformation of a substrate of interest is often challenging and time consuming. There are two strategies commonly used in enzyme discovery (Figure 1, left panel). One is to manually select the enzymes that have been reported to be active toward similar substrate(s) in the literature. However, this approach restricts the diversity of accessible enzymes and may result in potentially active enzymes being missed. Consequently, the alternative strategy of searching for homologous enzymes from databases is increasingly applied. However, this approach typically generates a very large number of sequences, the majority of which have not been characterized experimentally.^6,7^ Screening all of these sequences is time consuming, requiring the extensive use of analytical equipment and other methods for high-throughput screening.

Computational tools have shown great power in the development and application of biocatalysts including synthetic pathway design,^8−10^ enzyme engineering,^11−14^ and de novo design.^15−18^ However, in silico pipelines that prioritize sequences and thus reduce the necessary screening throughput in the laboratory are still underdeveloped. The current structure-based pipelines are either struggling in dealing with large numbers of sequences and are thus mainly used for engineering an initially known active enzyme^19,20^ or require a detailed description of the catalytic geometry based on a known ligand-binding motif.^21−23^ Sequence-based pipelines^24−38^ can realize high throughput but they generally ignore substrate variations (a key consideration in establishing biocatalytic panels), leading to poor activity predictions of enzymes in relation to target substrates. Standalone methods such as homology modeling and molecular docking have been established over many years but are generally used individually rather than in automated combination, which limits throughput in biocatalyst screening programs. Integration of these approaches into an automated pipeline for biocatalyst discovery is urgently needed to rapidly model enzyme–substrate complexes and generate rank ordering of likely active sequences. Only a few structure-based computational pipelines^38−41^ have been published. However, none of these have been automated to enable easy access to laboratory-based scientists. An “easy-to-use” in silico web application is required for experimentalists who need to conduct efficient and rapid screens to identify active enzymes but are struggling to use more specialized computational tools. Consequently, an “easy-to-use” in silico web-based application will streamline experimental screening and thereby reduce the time and resources required to identify biocatalysts for use.

**Figure 2:**
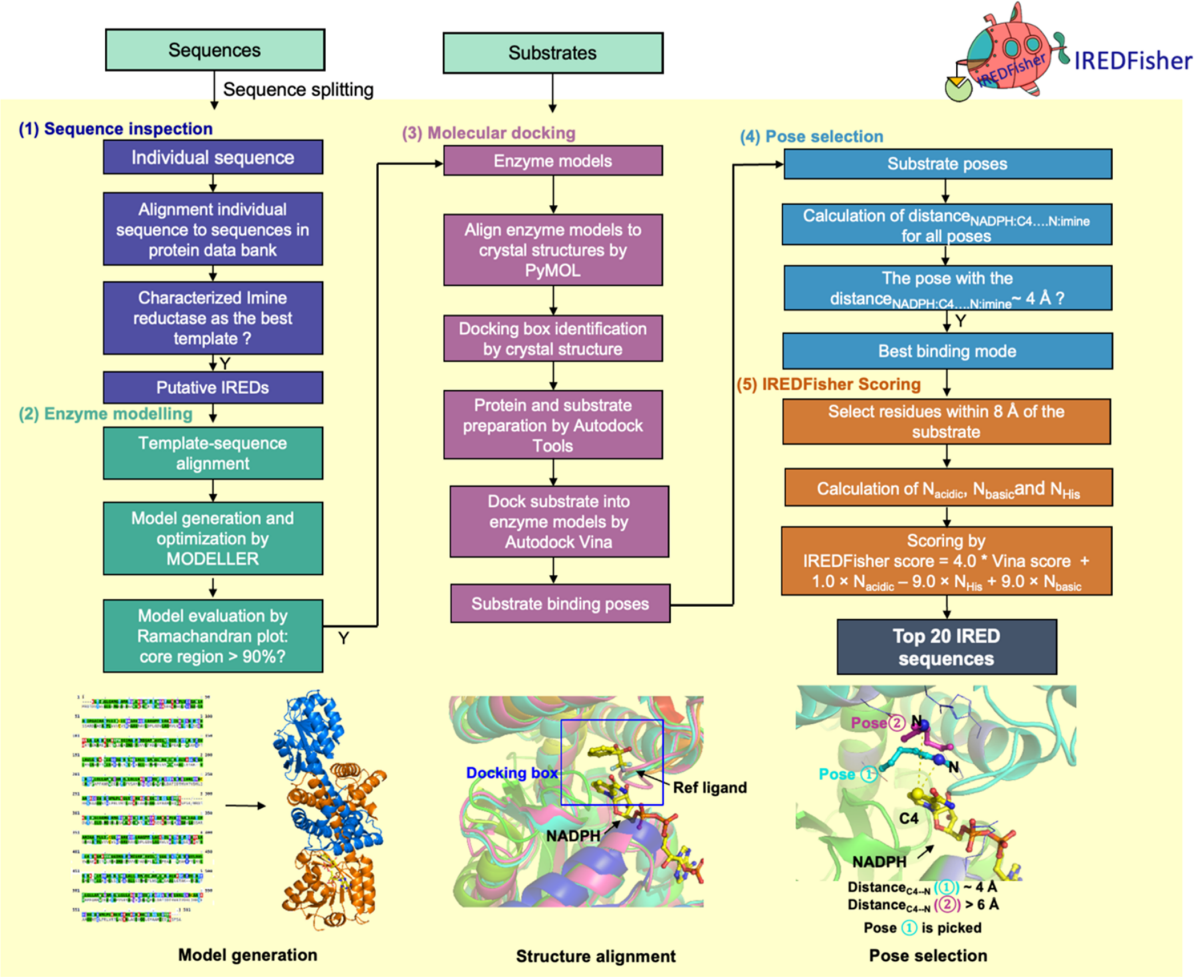
Flow chart of the four-step IREDFisher workflow. The input sequences are preprocessed in step (1) to remove non-IRED-homologs from the screening list. The three-dimensional structures of the remaining sequences are modeled in step (2). In step (3), the structures obtained from step (2) are aligned to a cofactor- and ligand-bound IRED crystal structure to locate the active site and cofactor binding site for each modeled structure. NADPH is placed into individual modeled structures by taking the coordinates from the crystal structure. An intermediate imine structure formed by amine and ketone substrates is then docked into the active site of each structure. In step (4), the best pose for the substrate within the enzyme is selected by the distance between two key atoms of the cofactor and substrate. In crystal structures of IREDs, this distance is around 4 Å. In step (5), the number of acidic residues *N*_acidic_, the number of basic residues *N*_basic_, and the presence of histidine residues *N*_his_ = 0 or 1 are calculated for residues within 8 Å of the substrate. Enzymes are ranked by a modified scoring function IREDFisher score = 4.0 × Vina score + 1.0 × *N*_acidic_ – 9.0 × *N*_His_ + 9.0 × *N*_basic_ (see details described in the Methods section).

Here, we report the development and experimental validation of an in silico pipeline, available also via a web interface, termed IREDFisher (available for use at https://enzymeevolver.com/IREDFisher). IREDFisher is a structure-based web server that allows computer-aided enzyme screening with substrate(s) of interest and recommends a small (20 enzyme) panel of IREDs for rapid biocatalytic exploration by the user (Figure 2). We selected imine reductases (IREDs) as a model enzyme in view of their recent emergence as an enzyme class of interest for the synthesis of chiral amines. Chiral amines are prevalent motifs that feature in about 40% of pharmaceuticals.^42,43^ IREDs catalyze enantioselective imine reduction and can also perform the reductive amination of ketones or aldehydes^44−46^ and are thus of major interest to the academic and industrial biocatalysis communities. Since their discovery in 2010,^47^ a vast number of IRED sequences have been published together with a wealth of activity data for different substrates.^48−58^ Currently, IREDFisher only applies to IREDs as the scoring function is modified specifically for these enzymes.

## Results

### IREDFisher Workflow

Having the initial sequences and a substrate structure as inputs, the IREDFisher workflow commences with sequence preprocessing, followed by structural modeling. Then, the provided substrate is docked into the modeled enzyme structures, while the protein–substrate complexes are scored by considering predicted binding affinity and catalytic geometry in the final step (Figure 2). Specifically, in step (1), each input sequence is aligned to individual sequences in the protein data bank (PDB). The input sequence that has the highest sequence identity to known IREDs in the PDB is considered as a putative IRED and retained for the next step. In step (2), all three-dimensional structures of the resulting sequences from step (1) are generated by MODELLER^59^ followed by an optimization by the default function in MODELLER,^59^ which consists of spatial restraints on distance and dihedral angles obtained from the target structure and CHARMM energy terms enforcing proper stereochemistry.^60^ All models are evaluated based on the Ramachandran plot, and only those that have over 90% of the residues in favorable/coreregions are defined as qualified models and subsequently used for substrate docking. In step (3), the substrate is docked into the active site of each qualified model. Various poses of the substrate in the active site of the enzymes are generated. In step (4), the distance between NADPH C4 and N atom of the imine substrate is calculated for all poses. The pose with ∼4 Å is selected as the best pose to realize hydride transfer in a catalytic process (see pose selection in Figure 2 and details in the Methods section). In step (5), the number of acidic residues *N*_acidic_, basic residues *N*_basic_, and histidine residues *N*_His_ are calculated and used to modify the Autodock Vina^61^ scoring function considering they play important roles in the stabilization of iminium intermediate formation and proton transfer (see details in the Methodssection). The modified scoring function Refined score = 4.0 × Vina score + 1.0 × *N*_acidic_ – 9.0 × *N*_His_ + 9.0 × *N*_basic_ was used to finally rank all input sequences. The top 20 IRED sequences are then selected for in vitro screening.

### IREDFisher Validation Using Published Screening Data

**Figure 3:**
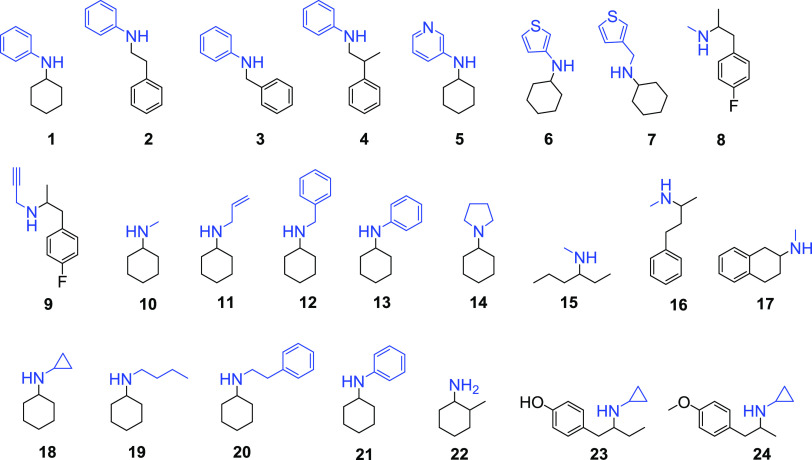
Tests of reductive amination reactions based on published data. Reaction products of IRED-catalyzed reactions are shown with moieties originating from the amine nucleophile colored in blue and those from the ketone component displayed in black. Compounds **1–9** are from ref (56), compounds **10–17** are from ref (52), and compounds **18–24** are from ref (53).

**Figure 4:**
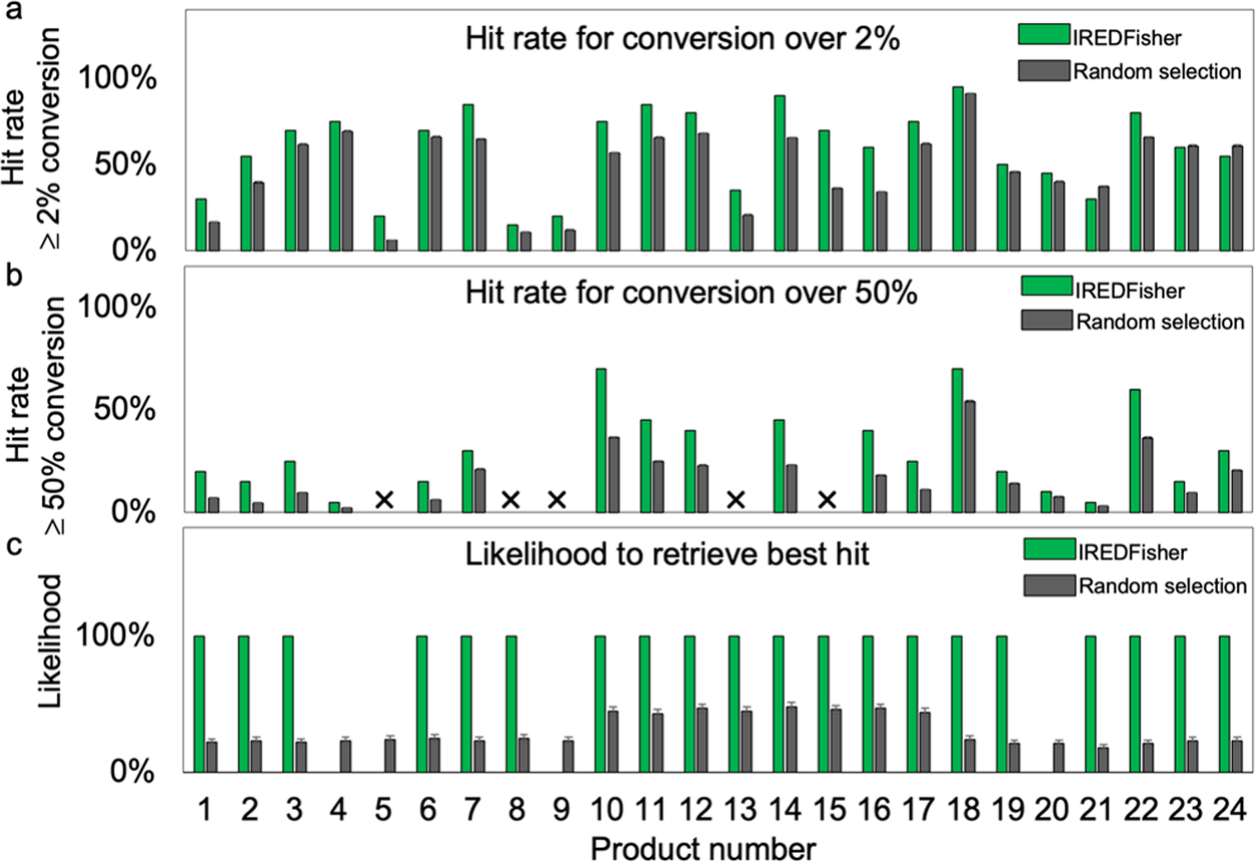
Comparison of hit rate and best hit retrieval in 20 sequences selected by IREDFisher and by random selection. (a) Hit rate using a conversion cutoff of 2% calculated by the equation: Hit rate of ≥2% conversion = . (b) Hit rate using a conversion cutoff of 50% calculated by the equation: Hit rate of ≥50% conversion = . Symbol × indicates that no hits with conversion over 50% were found in the in vitro screening of the corresponding panels. (c) Likelihood of retrieving the best hit from the whole panel in the 20 selected sequences using IREDFisher. The error bars for the random selection were calculated by the standard error of the mean (SEM) based on 95% confidence levels.

The screening data from three established IRED panels were used to test the IREDFisher workflow: Roiban et al. panel^56^ (85 sequences) for substrates **1**–**9**, France et al. panel^52^ (45 sequences) for substrates **10**–**17**, and Montgomery et al. IRED panel^53^ (93 sequences) for **18**–**24** (Figure 3). Each panel and the corresponding tested substrates were fed into IREDFisher to obtain the recommended 20 sequences. As control, 20 sequences were randomly selected from each corresponding panel. Hit rates using a conversion cutoff of 2% (hit rate of ≥ 2% conversion) and 50% (hit rate of ≥ 50% conversion) were calculated and compared between the two methods (Figure 4a,b and Table S1). The likelihood of retrieving the best hit in the 20 selected sequences using the two methods was also calculated (Figure 4c and Table S2). In terms of “hit rate of ≥ 2% conversion”, IREDFisher is comparable to random selection, whereas it outperforms the latter in finding hits with conversions over 50% (Figure 4b). This shows the capability of IREDFisher in finding competent hits for downstream optimization. Furthermore, IREDFisher retrieved the best hit to the top 20 sequences with a likelihood of 100%. By contrast, the likelihood of picking the best hit by random selection is no more than 50%. In summary, the IREDFisher workflow is not only able to improve the hit rate but also retrieves the best enzyme(s) from the whole IRED panel in the top 20 selected sequences. This emphasizes the effectiveness of IREDFisher in finding IRED hits, reducing screening efforts by 50–75%.

### Experimental Validation of Predictions Made by IREDFisher

**Figure 5:**
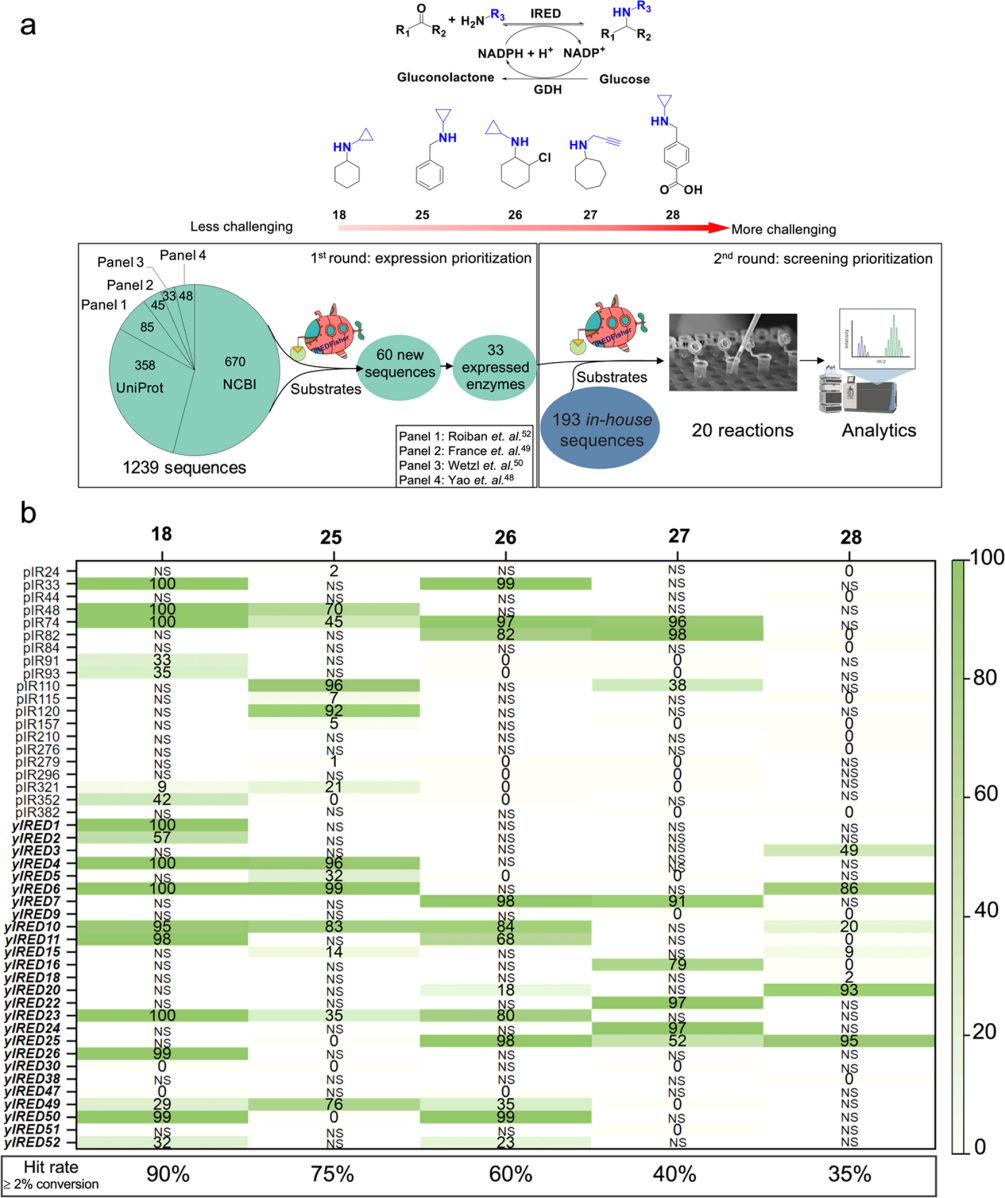
Validation of IREDFisher predictions in identifying hits for five chosen reactions. (a) IREDFisher was run in two rounds: in the first round, the sequences from external sources, public databases, and established panels were ranked by IREDFisher to prioritize those to be used for gene synthesis and enzyme expression. In the second round, 33 new enzymes with good expression in *Escherichia coli* and 193 in-house sequences were ranked by IREDFisher. The top 20 enzymes for each reaction were then investigated in the laboratory. The percentages of new enzymes and in-house enzymes are 65 and 35% for **18**, 50 and 50% for **25**, 45 and 55% for **26**, 50 and 50% for **27**, and 55 and 45% for **28**. Compounds are shown in product forms. Amine nucleophile components are colored in blue. (b) The conversion of IREDFisher selected sequences toward products **18** and **25**–**28**. Hit rates for each reaction were calculated using a conversion cutoff of 2%: Hit rate = . NS indicates that this sequence is not in the top 20 selected by IREDFisher and consequently was not tested in vitro. Sequences labeled pIR are from the in-house panel. Sequences labeled yIRED are new uncharacterized sequences and highlighted in bold and italic font.

Next, we challenged the IREDFisher workflow by aiming to select potential IRED hits for subsequent validation by experimental screening. We chose the following reductive amination reactions as our targets, namely, **18** and **25–28** (Figure 5a), which represent increasingly demanding conversions for imine reductases. Reactions corresponding to products **18** and **25** are common starting points for screening IREDs, whereas products **26** and **27** are important building blocks in the pharmaceutical industry and proved to be challenging targets in a previous IRED study.^48^ Synthesis of **28** is particularly challenging due to the negative charge of the carboxylate group in the substrate. To date, no IREDs have been reported to accept 4-formylbenzoic acid as a substrate. Also, we note that 4-formylbenzoic acid **28** is a building block for drugs such as procarbazine (chemotherapy), imatinib (chemotherapy), mocetinostat, etinostat, tucidinostat (HDAC inhibitors), revefenacin (COPD treatment), bavisant (ADHD treatment), and fedovapagon (nocturia treatment) (Figure S1). Inclusion of **28** was used to explore whether the IREDFisher workflow could identify biocatalysts for novel reductive amination reactions.

For sequence input, we used 193 well-expressed enzymes from our in-house panel along with 33 new sequences found by IREDFisher that are also expressed well. To find new active enzymes toward selected targets, we collected uncharacterized IRED homologs from public databases (NCBI and UniProt) and IREDs from established panels (Robian et al.,^56^ France et al.,^52^ Wetzl et al.,^54^ and Yao et al.^51^), generating 1239 sequences in total (Figure 5a, lower panel). Then, the first round of the IREDFisher ranking was run for the collected sequences based on the targeted product to prioritize successful gene synthesis and protein expression. The top 20 enzyme sequences for targets **18** and **25–30** were selected in silico. Then, 60 sequences were identified after removing duplicates from which genes were synthesized and expressed in *E. coli*. This resulted in 33 enzymes (55% of selected enzymes) that expressed well in *E. coli*, while 27 enzymes (45% of selected enzymes) failed in cloning and/or expression.

We then combined these 33 enzymes with 193 sequences from our in-house panel and ran a second round of IREDFisher ranking to prioritize these sequences in activity screening experiments. The top 20 IREDs obtained by IREDFisher for each targeted reaction were screened in vitro, and corresponding conversions to products were determined by GC or HPLC (Figure 5b). Reaction optimization was not carried out, as only the relative efficiency of IREDFisher under standardized conditions was being evaluated. For target reactions **18** and **25–28**, the hit rate decreases as the difficulty of the IRED reactions increases, with 2–11 hits (over 90% conversion) of corresponding substrates obtained. In a previous study, the hit rate of **26** (26b in a previous paper^48^) and **27** (**23d** in a previous paper^48^) by screening a panel of 384 IREDs was low (2.6% for **26** and 5.5% for **27**), whereas the hit rate in this study employing IREDFisher increased substantially to 60 and 40%, respectively. Notably, two hits, with conversion ≥ 90%, were found for the challenging product **28**, demonstrating that IREDFisher can find enzymes that catalyze reactions of pharmaceutical importance. Furthermore, by exploiting the resources from public databases, highly active IREDs that have not been reported previously were identified and multiple enzymes (e.g., yIRED10, yIRED23, yIRED25) were found to have broad substrate scope. The benefit of IREDFisher in prioritizing screening is clear, in this case reducing the experimental screening required from ≥ 1000 samples without IREDFisher virtual screening to only 20 samples.

### IREDFisher Web Interface and Speed Test

**Figure 6:**
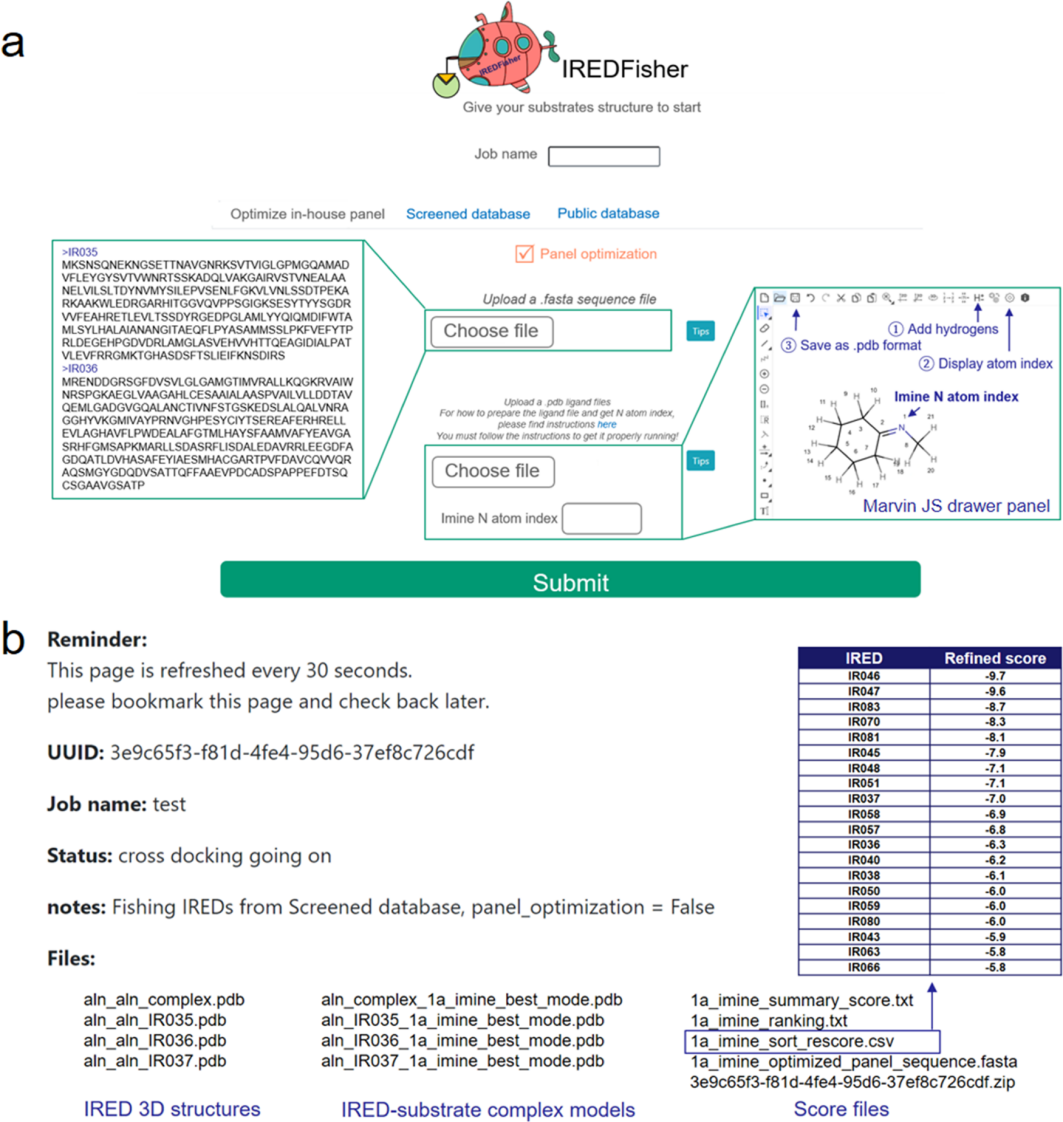
Web interface of IREDFisher. (a) Job submission interface and input file examples. Left square: FASTA-formatted sequence file. Right square: generation of substrate structure and N atom index by Marvin JS (<underline≥https://marvinjs-demo.chemaxon.com/latest/</underline≥). (b) Output files. The file for sequence ranking (1a_imine_sort_rescore.csv in the example) is shown.

Encouraged by the validation results using established screening data and our own experiments, our intention was to develop a user-friendly workflow that can be widely used by the biocatalysis community. To avoid any requirement for the use of personal computational/programming skills, we have built an IREDFisher web server (https://enzymeevolver.com/IREDFisher) (Figure 6). We emphasize that the current methodology only applies to IREDs. For the input structure, the imine structure formed by the condensation of aldehyde or ketone and amine is required. This can be obtained by the free online structure drawing tool Marvin JS (https://marvinjs-demo.chemaxon.com/latest/) from ChemAxon (Figure 6a, example in the square on the right). The index of the N atom in the C=N bond is also required as an input, which can be generated using the Marvin interface. A FASTA-formatted sequence file is required if users intend to rank their own sequences (Figure 6a, example in the square on the left). After the job is submitted, output files such as three-dimensional structures of IREDs, substrate-bound complex structures, and scoring files are generated and added to the web page. By checking the sorted rescoring file, the user can obtain an optimized small IRED screening panel with 20 recommended sequences by IREDFisher (Figure 6b). In addition, IREDFisher also allows users to rank the sequences from established panels and public databases (see the Methods section for details). Therefore, IREDFisher can be employed in ranking: (i) user-defined sequences; (ii) established IRED panels, e.g., Robian et al.,^56^ France et al.,^52^ Wetzl et al.,^54^ etc.; and (iii) IRED sequences collected from the public database in this study. The speed test showed that IREDFisher can perform the calculation of a user-defined panel with 85 sequences in 90 min. Moreover, it only takes ca 30 min to complete the ranking of the established IRED panels and sequences from public databases by using the structures already deposited in the IREDFisher web server. Consequently, based on simple data input, nonspecialists in bioinformatics can have access to workflow outputs using only a few mouse clicks without human interference, which makes it applicable for small IRED panel design.

## Discussion

The computational four-step workflow IREDFisher that aims to streamline the screening of enzyme panels was produced by integrating sequence analysis, structure modeling, and molecular docking. Although large numbers of protein structures have been released recently by AlphaFold,^62^ the structures obtained are not always applicable for the use of molecular docking. For many enzymes, the substrate-binding site is formed by monomers/dimers, but only monomeric structures were deposited in the database. For example, most of the new sequences used in this study were only modeled as monomer in the AlphaFold database. We have shown that the active site is similar between the dimer models generated by homology modeling and AlphaFold (Figure S2), while the former approach saves considerable computation time (3 min by homology modeling versus 30 min by AlphaFold per structure). This is also suggested by a previous publication.^63^ Therefore, the template-based homology modeling is beneficial for the rapid building of structures in the IREDFisher workflow, allowing users to rank large numbers of sequences.

The workflow was applied by using a model enzyme IRED and subsequently validated using previously published screening data. IREDFisher demonstrated the power of retrieving the best hit found simply by screening a small panel of 20 sequences using the IREDFisher workflow. Experimental validation in which IREDFisher selects 20 enzymes from a pool of ∼1400 sequences for 5 reactions, and with different levels of product complexity, further establishes the effectiveness of IREDFisher. IREDFisher ranks enzymes based on enzyme–substrate-binding interactions and does not consider features such as the charge distribution on the protein surface, which might, for example, be important for successful protein expression in *E. coli*. As such, poorly expressed proteins could rank better than proteins that are expressed well in *E. coli*. The workflow was run initially to identify sequences for gene synthesis and protein expression analysis. We then performed a second screening experiment to rank new IRED biocatalysts only with those sequences that expressed well in *E. coli*. The prediction of enantioselectivity by IREDFisher is not investigated in this study because this is challenging. That said, IREDFisher narrows the search to a small panel of IREDs such that screening those biocatalysts for enantioselectivity is no longer an onerous task.

It is noteworthy that most biocatalytic processes for drug production need to go through extensive reaction optimization and mutagenesis approaches such as directed evolution to obtain a biocatalyst that fulfills the desired process requirements. Directed evolution relies on the screening of thousands of enzyme variants and usually involves several rounds of library generation and screening, resulting in large numbers of variants that require high-throughput screening (HTS) methods for their evaluation. As such, screening remains the major bottleneck in biocatalysis applications, and this places major burdens on the resources and time required to generate a suitable biocatalyst. By taking advantage of the IREDFisher computational workflow, we have shown that it is possible to identify suitable biocatalysts for simple and challenging substrates. If needed, these could be evolved further to fulfill process needs with reduced screening efforts. IREDFisher is therefore valuable, either as a standalone computational workflow to aid the rapid identification of biocatalysts or as a “front-end’ workflow to accelerate directed evolution programs by selecting the prototype, catalytically competent enzyme scaffolds for a target reaction. However, we clarify that IREDFisher takes wild-type sequences as input, which limits its ability to “design” enzymes with non-natural functions to catalyze extremely challenging reactions.

IREDFisher is designed as a “fast prescreen method” to rank input sequences. As such, it has limitations. Protein dynamics and energy barrier calculations are not incorporated because this would be too time consuming for a fast prescreening tool for use by experimentalists. The IREDFisher scoring function works as a tool to rank input sequences instead of accurately predicting the conversion/activity of enzymes. We trained the scoring instead to deliver a maximum hit rate of the top 20 sequences because 20 screening experiments can be carried out in one batch with overnight incubation for rapid enzyme screening. That said, the IREDFisher workflow has the power to find highly active hits in the 20 recommended sequences. This indicates that a trade-off between speed and accuracy has been achieved to some extent. Currently, it is applicable to IREDs as the scoring function is designed specifically for this enzyme family. We have also tested IREDFisher on haloalkane dehydrogenases and compared our results with a previous study.^39,40^ Only one enzyme identified by this previous study was ranked in the top 20 sequences from IREDFisher. Clearly, generic substrate inputs and associated scoring functions will need to be explored to expand the utility of our workflow to different enzyme reactions. Specifically, reactive atoms in the substrate and catalytic residues in the enzyme should be provided by the user to help select the best binding pose after docking. Also, deep learning methods are promising approaches to train a model, e.g., by taking features from predicted binding modes such as distances and angles between key atoms, atom types, and charge distributions in the active site. This challenge, together with integration into RetroBiocat,^8^ will be implemented to enable the process from the biocatalytic pathway design all the way through to the generation of small screening panels to allow for direct hit identification.

In summary, the development and use of IREDFisher have reduced the size of screening panels to only 20 enzymes, with significant savings in resource and time required to perform the experimental screening. For challenging substrates, where conversions with the wild-type enzyme may be low, the hits obtained could then be used as a parent sequence for subsequent directed evolution approaches. The workflow provides a user-friendly free web interface (https://enzymeevolver.com/IREDFisher), requiring no experience in computational chemistry or programming, which users can implement with only a few “mouse clicks”. The expansion of the web tool to other enzyme classes is envisaged with more accurate scoring functions, taking advantage of machine learning and other approaches. This will assist in the identification of enzymes for transformations of interest from a plethora of continuously growing sequence data and, therefore, contribute to the increasing application of biocatalytic methods in the synthesis.

## Methods

### IREDFisher Four-Step Panel Optimization Workflow

#### Sequence Preprocessing

A FASTA-formatted sequence file given by the user was loaded into the workflow. Each individual sequence was generated by splitting the whole sequences. For sequence, we searched homologous proteins in the Protein Data Bank using MODELLER.^59^ The one with the highest sequence identity was considered as the template protein of the query sequence. For imine reductases, only sequences with characterized IREDs (PDB codes: 3ZGY,^64^4D3D,^65^4D3S,^65^4OQY,^66^4OQZ,^66^5A9T,^67^5OCM,^68^5OJL,^69^6EOD,^44^6JIT, 6JIZ, 6GRL, and 5G6R) as the template proteins were retained for the next step.

#### Structure Modeling

IREDs form homodimers with the active site located at the interface between the monomers, thus making it more challenging to build a model in an automated way.^70^ The general sequence alignment between the template protein and query sequence was calculated only for one chain. To overcome this issue, the single-chain sequence alignment between each query sequence and the template protein was repeated once more by an in-house script to generate a double-chain sequence alignment file. To automate the modeling of the IRED dimeric form, a template protein needs to be specified. The imine reductase from *Streptosporangium roseum* (PDB code: 5OCM(68)) is selected as the dimer template protein given its high resolution and the clear substrate and cofactor NADPH binding site. Then, models are generated and optimized using MODELLER^59^ based on the modified double-chain sequence alignment. All models are evaluated based on the Ramachandran plot, and only those that have over  90% of the residues in the favorable/core region are defined as qualified models and subsequently are used for substrate docking.

#### Molecular Docking

To locate the active site easily, all three-dimensional structures obtained from the last step are aligned to the template structure 5OCM by the PyMol python library. The cofactor NADPH is directly placed into the cofactor binding site of each modeled structure using the reference NADPH from the crystal structure prior to substrate docking. The center of mass of the bound ligand 9RH in 5OCM is used as the center of the docking box. The docking box size is calculated by using script eBoxSize.pl,^71^ considering the gyration radius of the substrate, which improves the docking accuracy.^71^ Then, all aligned structures with the bound NADPH are prepared using AutoDockTools.^72^ To input the structure of the imine form of the substrate formed by the condensation of amine and ketone, the three-dimensional coordinates are generated by Babel^73^ and then prepared using AutoDockTools.^72^ Multiple poses are generated by Autodock vina^61^ with exhaustiveness value 10.

Scoring and ranking. When the IRED reaction occurs, NADPH transfers a hydride from atom C4 to the imine substrate.^44,70,74^ To obtain the most reliable pose, the distance between the C4 atom in NADPH and N in imine, the substrate is used as a criterion (Figure 2). Docking poses with the C4···N distance shorter than 3.5 Å are removed to avoid clashes. Poses with the C4···N distance longer than 6.0 Å are also removed because it is beyond the distance range for hydride transfer. The remaining poses having the closest C4···N distance are selected as the best model. To improve the scoring function, we take the catalytic mechanism, proton transfer, and hydride transfer^75^ into consideration. It has been reported that acidic residues, basic residues, and a conserved histidine residue in the active site play important roles in the stabilization of the key intermediate in the IRED-catalyzed reaction.^75^ Accordingly, we included three terms in the scoring function: the number of acidic residues *N*_acidic_, the number of basic residues *N*_basic_, and the presence of histidine residues to represent the effect enzymes have on reactions. The final scoring function after the optimization of hyperparameters is refined score = 4.0 × Vina score + 1.0 × *N*_acidic_ – 9.0 × *N*_His_ + 9.0 × *N*_basic_. In this equation, *N*_acidic_/*N*_basic_ is the total number of acidic/basic residues in the active site and *N*_His_ is the presence (*N*_His_ = 1) or absence of histidine (*N*_his_ = 0) residue in the active site. The weights for each component of the scoring function were tuned by running with a range of −10 to 10 using compounds **18**–**24** screened by Montgomery et al. IRED panel,^53^ and the best weight was decided by the highest percentage of hits with conversion over 50% in the top 20 list. The scoring function was tested by Roiban et al. panel^56^ for substrates **1**–**9** and France et al. panel^52^ for substrates **10**–**17**. Only residues within 8 angstroms of the ligand are taken into account when we count the number of key residues.

### IREDFisher Structure Databases

To help users design an IRED screening panel from scratch by the IREDFisher workflow, we collected published IRED panels: GSK panel in 2017,^56^ MIB panel in 2018,^52^ MIB panel in 2020,^53^ MIB metagenomic panel in 2021,^48^ Roche panel in 2016,^55^ and Peiyuan Yao panel^51^ in 2018. After sequence preprocessing step, a total of 451 published sequences were collected including the characterized IRED sequences.

To make use of the public database, we conducted homology search in UniProt^6^ and NCBI reference protein^76^ databases using the characterized IREDs as seed sequences. Homologous sequences with sequence identity over 30%, coverage over 80%, and *E*-value under 10 compared to the corresponding seed sequence were downloaded. 1028 sequences were collected after removing duplicates, followed by a clustering with threshold 0.7 by cd-hit.^77^ After sequence preprocessing, a total of 591 sequences were collected from the public database.

All of the above sequences were modeled and prepared for docking by the IREDFisher workflow. With the well-prepared structure database, IREDFisher runs much faster.

## Experimental Details for In Vitro Screening

### General (Chemicals and Enzymes)

All chemicals and solvents were purchased from commercial sources such as Acros Organics (Geel, Belgium), Fluorochem (Hadfield, Derbyshire, UK), Alfa Aesar (Karlsruhe, Germany), and Sigma-Aldrich (Poole, Dorset, UK). Chemicals were used without additional purification. GC gases were obtained from BOC gases (Guildford, UK), and HPLC solvents were obtained from Honeywell (Seelze, Germany).

GC-MS analysis was performed using an Agilent 7890B Series GC with a 5977B MS-EI detector in positive mode at a constant He flow. HPLC analysis was performed using an Agilent 1200 Series with a UV detector Agilent 1200 Series. LC-MS analysis was performed using an Agilent 1260 Infinity II with an Agilent 6130 Quadrupole detector in positive mode using ESI as the ionization source.

Conversions were determined by achiral HPLC and GC-MS and calculated with respect to the carbonyl compound based on the convention used for reductive amination and by LC-MS with the calibration curve. Products were identified by LC-MS and by comparing retention time with standards.

## Enzyme Production and Expression

All IREDs used in this study were obtained and expressed using the same conditions as outlined in https://doi.org/10.1038/s41557-020-00606-wSupporting Section S3.

## Screening

### Enzyme Screening for Target Product **18**

Analytical scale reductive amination biotransformations were carried on 500 μL scale, adjusted to pH 8.0, containing 4 mg/mL of lyophilized powder of the supernatant of lysate IRED, 0.5 mg/mL of GDH (Codexis CDX-901), 0.5 mM of NADP^+^ (Prozomix), 20 mM of d-glucose, 2.5% of DMSO, 5 mM of cyclohexanone, and 10 equivalents of cyclopropylamine (1 M cyclopropylamine stock pH 8.0 adjusted), with the reaction volume made up to 500 μL in 100 mM tris buffer. The reaction mixture was incubated at 37 °C with shaking at 900 rpm (Eppendorf ThermoMixer) for 20 h. After 24 h, 500 μL was recovered from the reaction mixture and quenched with 20 μL of 10 M sodium hydroxide and extracted with 500 μL of dichloromethane (DCM). The organic phase was dried over anhydrous magnesium sulfate (MgSO_4_) and analyzed by GC-MS.

## Enzyme Screening for Target Product **25**

Analytical scale reductive amination biotransformations were carried on a 500 μL scale adjusted to pH 8.0 containing 4 mg/mL of lyophilized powder of the supernatant of lysate IRED, 0.5 mg/mL of GDH (Codexis CDX-901), 0.5 mM of NADP^+^ (Prozomix), 40 mM of d-glucose, 5% of DMSO, 10 mM of benzaldehyde, and 5% and 10 equivalents of cyclopropylamine (1 M cyclopropylamine stock pH 8.0 adjusted), with the reaction volume made up to 500 μL in 100 mM tris buffer. The reaction mixture was incubated at 37 °C with shaking at 900 rpm (Eppendorf ThermoMixer) for 24 h. Following 24 h, the reaction was quenched by the addition of 1 volume of methanol. The mixture was centrifuged for 5 min at 13300 rpm, and the supernatant was recovered to be analyzed by HPLC and LC/MS.

## Enzyme Screening for Target Product **26**

Analytical scale reductive amination biotransformations were carried out on a 500 μL scale adjusted to pH 8.0 containing 4 mg/mL of lyophilized powder of the supernatant of lysate IRED, 0.5 mg/mL of GDH (Codexis CDX-901), 0.5 mM of NADP^+^ (Prozomix), 20 mM of d-glucose, 5 mM of 2-chlorocyclohexanone, and 10 equivalents of cyclopropylamine (1 M cyclopropylamine stock pH 8.0 adjusted), with the reaction volume made up to 500 μL in 100 mM tris buffer. The reaction mixture was incubated at 37 °C with shaking at 900 rpm (Eppendorf ThermoMixer) for 24 h. After 24 h, 250 μL was recovered from the reaction mixture and quenched with 20 μL of 10 M sodium hydroxide and extracted with 250 μL of dichloromethane (DCM) to extract the substrate and possible byproducts. The organic phase was dried over anhydrous magnesium sulfate (MgSO_4_). The other 250 μL was extracted with 250 μL of dichloromethane (DCM) to extract the product. The two organic phases were combined, dried over anhydrous magnesium sulfate (MgSO_4_), and analyzed by GC-MS.

## Enzyme Screening for Target Product **27**

Analytical scale reductive amination biotransformations were carried out on a 500 μl scale adjusted to pH 8.0 containing 4 mg/mL of lyophilized powder of the supernatant of lysate IRED, 0.5 mg/mL of GDH (Codexis CDX-901), 0.5 mM of NADP^+^ (Prozomix), 40 mM of d-glucose, 5% of DMSO, 10 mM of cycloheptanone, and 10 equivalents of propargylamine (1 M propargylamine stock pH 8.0 adjusted), with the reaction volume made up to 500 μL in 100 mM tris buffer. The reaction mixture was incubated at 37 °C with shaking at 900 rpm (Eppendorf ThermoMixer) for 24 h. After 24 h, 500 μL was recovered from the reaction mixture and quenched with 20 μL of 10 M sodium hydroxide and extracted with 500 μL of dichloromethane (DCM). The organic phase was dried over anhydrous magnesium sulfate (MgSO_4_) and analyzed by GC-MS.

## Enzyme Screening for Target Product **28**

Previous test reactions were carried on at pH 8 as IREDs usually exhibit higher activities on slightly basic pH. However, reaction test 3 was done at pH 6 since the acid is partially deprotonated and the aim was to use pH values closer to formylbenzoic acid pKa to make it less negatively charged. Analytical scale reductive amination biotransformations were carried out on a 500 μL scale adjusted to pH 6.0 containing 4 mg/mL of lyophilized powder of the supernatant of lysate IRED, 0.5 mg/mL of GDH (Codexis CDX-901), 0.5 mM of NADP^+^ (Prozomix), 20 mM of d-glucose, 2.5% of DMSO, 5 mM of 4-formylbenzoic acid, and 20 equivalents of cyclopropylamine (1 M cyclopropylamine stock pH 6.0 adjusted), with the reaction volume made up to 500 μL min of 100 mM 2-(*N*-morpholino) ethanesulfonic acid (MES) buffer. The reaction mixture was incubated at 37 °C with shaking at 200 rpm (incubator with orbital shaker) for 24 h and quenched by the addition of 1 volume of methanol. The mixture was centrifuged for 5 min at 13 300 rpm, and the supernatant was recovered to be analyzed by HPLC and LC/MS.

## Data Availability

All data are within the article and the Supporting Information and are also available from the corresponding author upon reasonable request. IREDFisher is a free web server: https://enzymeevolver.com/IREDFisher. The source code was provided on GitHub (https://github.com/yuyuqi-design/iredfisher_src) and can be freely downloaded.

## Supporting Information Available

Additional in silico screening details; experimental details; and materials and methods, including HPLC and LC/MS chromatograms for the reactions conducted in this study and the sequences of all new putative imine reductases (PDF)
