# Supplementary material for: Structure-Based Design of Small Imine Reductase Panels for Target Substrates
Source: ACS Catal. 2023 Sep 5;13(18):12310–21. doi: 10.1021/acscatal.3c02278 (PMC10510103; doi:10.1021/acscatal.3c02278)
Supplement: Supplementary file 1 — cs3c02278_si_001.pdf [file cs3c02278_si_001.pdf]

Supplementary information

Structure-based design of small imine reductase panels for target substrates

Yuqi Yu[a,b,*], Arnau Rué Casamajo[a], William Finnigan[a], Christian Schnepel[a], Rhys Barker[a], Charlotte Morrill[a], Rachel S. Heath[a], Leonardo De Maria[c], Nicholas J. Turner[a,*], Nigel S. Scrutton[a,*]

[a]Department of Chemistry, The University of Manchester, Manchester Institute of Biotechnology, 131 Princess Street, Manchester, M1 7DN, UK.

[b]Augmented Biologics Discovery & Design, Department of Biologics Engineering, BioPharmaceuticals R&D, AstraZeneca, Cambridge, CB21 6GH, UK.

[c]Medicinal Chemistry, Research and Early Development, Respiratory and Immunology (RI), BioPharmaceuticals R&D, AstraZeneca, Gothenburg 43150, Sweden

[*]Email: nigel.scrutton@manchester.ac.uk; nicholas.turner@manchester.ac.uk; yuqi.yu@manchester.ac.uk;

Table of Contents

Table S1. Hit rate in 20 sequences by IREDFisher and random selection 3

Table S2. Likelihood to retrieve the best hit by IREDFisher and Random selection 4

Figure S1. Examples of pharmaceutically relevant 4-formylbenzoic acid benzaldehyde derivatives 5

Figure S2. Comparison of imine reductase structures predicted by homology modelling and AlphaFold. 6

Analytical methods 7

Figure S3. Scheme of reductive amination of cyclohexanone with cyclopropylamine for the synthesis of **18** 8

Figure S4. GC-MS spectra from screening for the synthesis of **18** 9

Table S3. Retention times from GC-MS analysis for test reaction **18** 9

Figure S5. Scheme of reductive amination of chlorocyclohexanone with cyclopropylamine for the synthesis of **26** 10

Figure S6. GC-MS spectra from screening for the synthesis of reaction **26** 11

Table S4. Retention times from GC-MS analysis for test reaction **26** 11

Figure S7. Scheme of reductive amination of cycloheptanone with propargylamine for the synthesis of **27** 12

Figure S8. GC-MS spectra from screening for the synthesis of **27** 13

Table S5. Retention times from GC-MS analysis for test reaction **27** 13

Figure S9. Scheme of reductive amination of benzaldehyde with cyclopropylamine for the synthesis of **25** 14

Figure S10. LC/MS chromatograms from screening for the synthesis of **25** 14

Table S6. Retention times from HPLC analysis for test reaction **25** 15

Figure S11. Scheme of reductive amination of 4-formylbenzoic acid with cyclopropylamine for synthesis of **28** 16

Figure S12. HPLC chromatograms from screening for the synthesis of compound **28** 16

Table S7. Retention times from HPLC analysis for test reaction **28** 17

IRED Sequences 17

Table S1. Hit rate in 20 sequences by IREDFisher and random selection. As a control, 20 sequences were randomly selected from the panels 1000 times and the average hit rate was calculated. The margin of error was calculated based on 95% confidence levels.

Reaction	Hit rate by IREDFisher (Conv. ≥50%)	Hit rate by random selection (Conv. ≥50%)	Improvement by IREDFisher[2] (Conv. ≥50%)	Hit rate by IREDFisher (Conv. ≥2%)	Hit rate by random selection (Conv. ≥2%)	Improvement by IREDFisher[2] (Conv. ≥2%)
1	20%	7% ± 0.3%	1.90	30%	17% ± 0.4%	0.82
2	15%	5% ± 0.2%	2.33	55%	40% ± 0.6%	0.39
3	25%	9% ± 0.3%	1.66	70%	62% ± 0.6%	0.14
4	5%	2% ± 0.2%	1.27	75%	69% ± 0.6%	0.08
5	0%	0% ± 0.0%	NA	20%	6% ± 0.3%	2.33
6	15%	6% ± 0.3%	1.46	70%	66% ± 0.6%	0.06
7	30%	21% ± 0.5%	0.43	85%	65% ± 0.6%	0.32
8	NA[1]	NA[1]	NA[1]	15%	11% ± 0.4%	0.42
9	NA[1]	NA[1]	NA[1]	20%	12% ± 0.4%	0.69
10	70%	36% ± 0.5%	0.92	75%	57% ± 0.5%	0.32
11	45%	25% ± 0.5%	0.82	85%	66% ± 0.5%	0.30
12	40%	23% ± 0.4%	0.75	80%	68% ± 0.5%	0.17
13	NA	NA[1]	NA[1]	35%	21% ± 0.4%	0.70
14	45%	23% ± 0.4%	0.96	90%	66% ± 0.5%	0.37
15	NA	NA[1]	NA[1]	70%	36% ± 0.5%	0.94
16	40%	18% ± 0.4%	1.22	60%	34% ± 0.5%	0.76
17	25%	11% ± 0.3%	1.25	75%	62% ± 0.5%	0.21
18	70%	54% ± 0.6%	0.30	95%	91% ± 0.3%	0.04
19	20%	14% ± 0.4%	0.43	50%	46% ± 0.6%	0.10
20	10%	8% ± 0.3%	0.33	45%	40% ± 0.6%	0.13
21	5%	3% ± 0.2%	0.61	30%	37% ± 0.6%	-0.19
22	60%	36% ± 0.6%	0.66	80%	66% ± 0.6%	0.22
23	15%	9% ± 0.4%	0.60	60%	61% ± 0.6%	-0.01
24	30%	20% ± 0.5%	0.47	55%	61% ± 0.6%	-0.09

[1] No hits with conversion over 50% were found in the overall screening.

[2] $$Improvement\ by\ IREDFisher = \frac{Hit\ rate\ by\ IREDFisher - Hit\ rate\ by\ random\ selection}{Hit\ rate\ by\ random\ selection}$$

Table S2. Likelihood to retrieve the best hit by IREDFisher and Random selection. In most cases, IREDFisher was able to retrieve the best hit(s) from the whole panel. In some cases where the best hit did not appear, the second best hit was found. By comparison, random selection has a much lower chance of obtaining the best hit(s).

Reaction	Best hit	IREDFisher	Random selection
1	IR-10	Yes: Rank **10**th	22% ± 2.6%
2	IR-01	Yes: Rank **9**th	23% ± 2.6%
3	IR-01	Yes: Rank **10**th	22% ± 2.6%
4	IR-13	No, IR-10 Rank **11**th	23% ± 2.6%
5	IR-01	No, IR10 Rank 10th	24% ± 2.6%
6	IR-01	Yes: Rank **9**th	25% ± 2.7%
7	IR-01	Yes: Rank **12**th	23% ± 2.6%
8	IR-01	Yes: Rank **16**th	25% ± 2.7%
9	IR01	No, IR-59 Rank **12**th	23% ± 2.6%
10	IR81	Yes: Rank **7**th	45% ± 3.1%
11	IR44	Yes: Rank **19**th	43% ± 3.1%
12	IR56	Yes: Rank **7**th	47% ± 3.1%
13	IR47	Yes: Rank **16**th	45% ± 3.1%
14	IR44	Yes: Rank **8**th	48% ± 3.1%
15	IR50	Yes: Rank 1**3**th	46% ± 3.1%
16	IR47	Yes: Rank **3**th	47% ± 3.1%
17	IR81	Yes: Rank **10**th	44% ± 3.1%
18	p-IR33	Yes: Rank **1**th	24% ± 2.6%
19	p-IR82	Yes: Rank **8**th	21% ± 2.5%
20	p-IR23	No; p-IR16 Rank **19**th	21% ± 2.5%
21	p-IR23	Yes: Rank **5**th	18% ± 2.4%
22	p- IR49	Yes: Rank **1**th	21% ± 2.5%
23	p-IR23	Yes: Rank **1**th	23% ± 2.6%
24	p-IR49	Yes: Rank **3**th	23% ± 2.6%

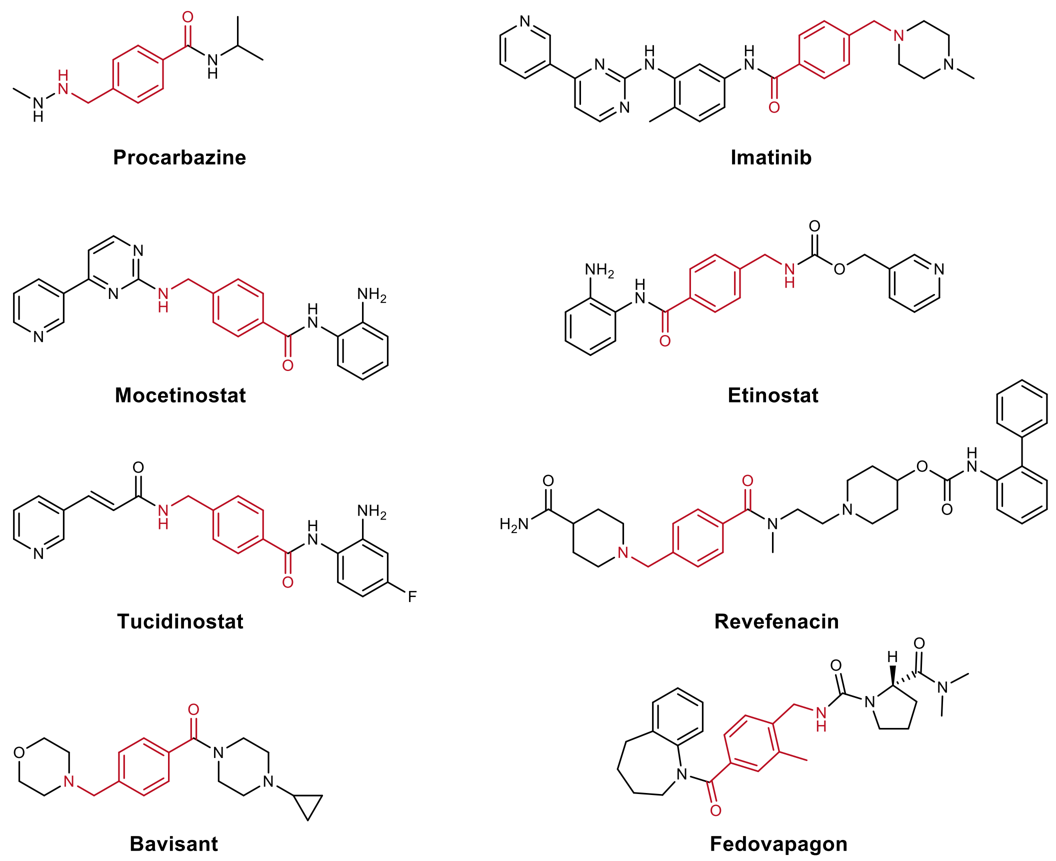

Figure S1. Examples of pharmaceutically relevant 4-formylbenzoic acid benzaldehyde (highlighted in red) derivatives.

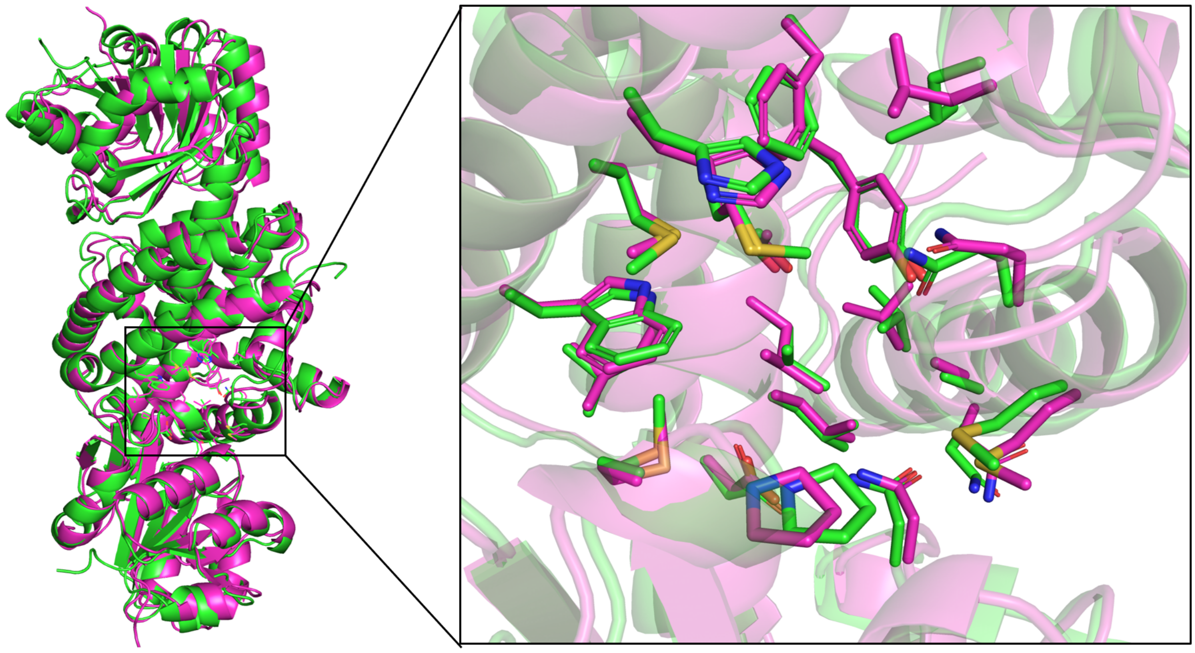

Figure S2. Structural comparison of an imine reductase predicted by homology modelling (in magenta) and AlphaFold (in green). Left panel, the overall structure. Right panel, a close-up view of amino acids in the substrate-binding site. Side chains in the active site were aligned, resulting in a root mean square deviation about 1.02 Å.

Analytical methods

GC-MS

Test reaction **18, 26** and **27**. The mixture was analysed by GC-MS using the following conditions: hold at 40°C for 3 minutes, 40-300°C (30 °C / min) and 0.5 minutes for equilibration. Injector temperature 270 °C; inlet pressure 5.34 psi; detector temperature 300 °C; helium flow 2 ml/min; transfer line 320 °C, quadrupole 150 °C, ion source 230 °C; column Agilent HP-1MS (30 m × 0.32 mm × 0.25 µm).

HPLC and LC/MS

Test reaction **25** and **28**. The mixture was analysed by HPLC and corroborated by LC/MS using the following conditions: eluent MeCN/H_2O + 0.1% trifluoroacetic acid with a gradient method from 5% to 95% MeCN for 15 minutes. Flow 0.5 ml/min; column temperature 40 °C; injection volume 5 µl; UV detection at λ = 210 nm, column: Phenomenex Luna 3 µm C18(2) 100 Å.

Test reaction **18** scheme

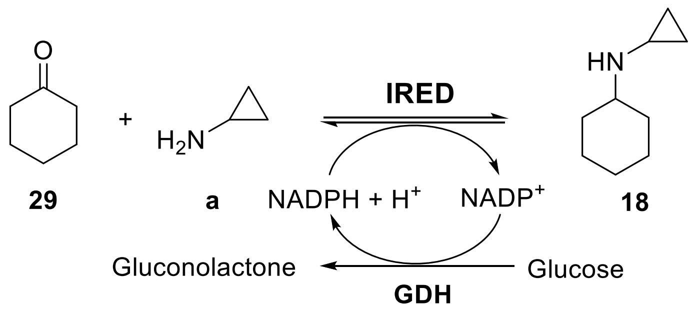

Figure S3. Scheme of reductive amination of cyclohexanone with cyclopropylamine for the synthesis of **18**. Cofactor regeneration system consists of GDH, $NADP^+$ and glucose.

Test reaction **18** chromatograms

a)
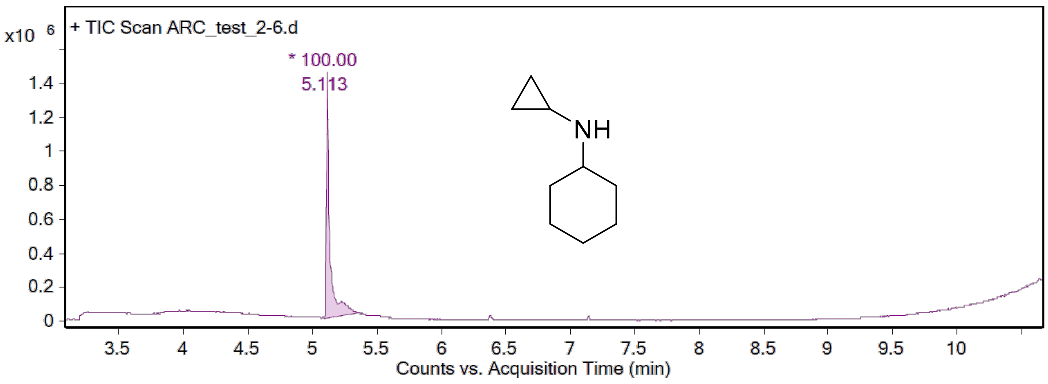

b)
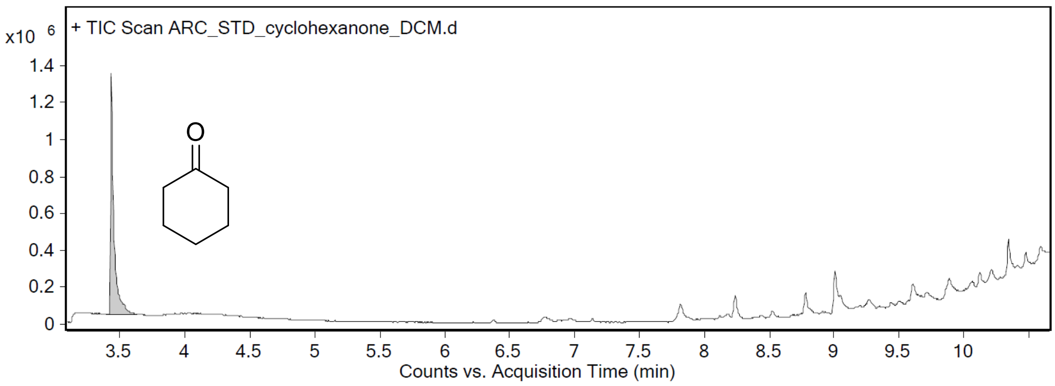

c)
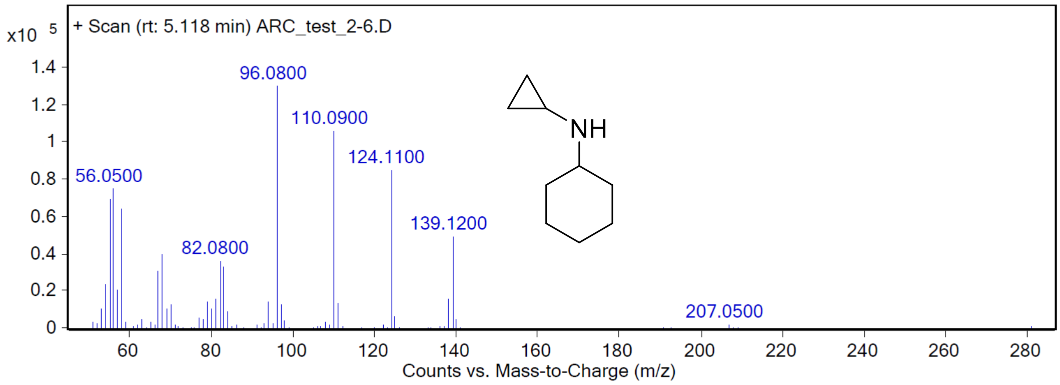

d) 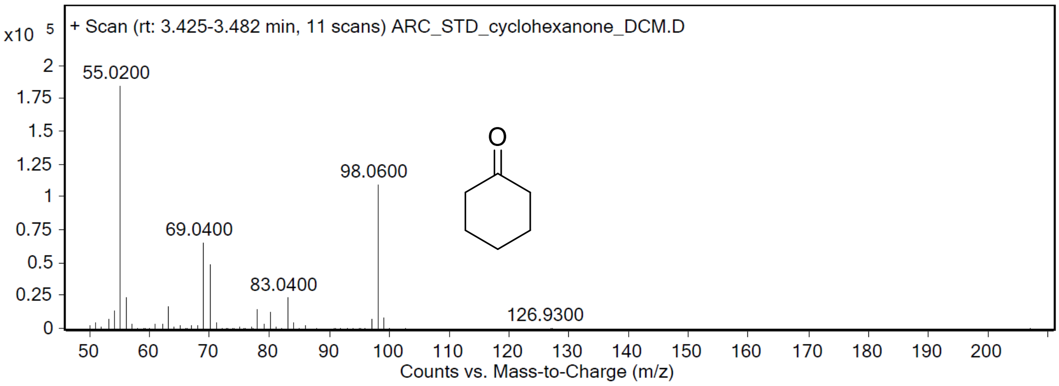

Figure S4. GC-MS spectra from screening for the synthesis of **18**. a) GC-MS chromatogram of cyclohexanone reductive amination with cyclopropylamine which exhibited >99% conversion. b) GC-MS chromatogram of cyclohexanone (**29**) standard. c) GC-MS spectra of peak at R_t = 5.11 min corresponding to GC-MS chromatogram a. d) GC-MS spectra of peak at R_t = 3.45 min corresponding to GC-MS chromatogram b.

Table S3. Retention times from GC-MS analysis for test reaction **18**.

Compound	Retention time (minutes)
*2-cyclohexanone (**29**)*	3.5
*N-cyclopropylcyclo hexanamine (**18**)*	5.1

Test reaction **26** scheme

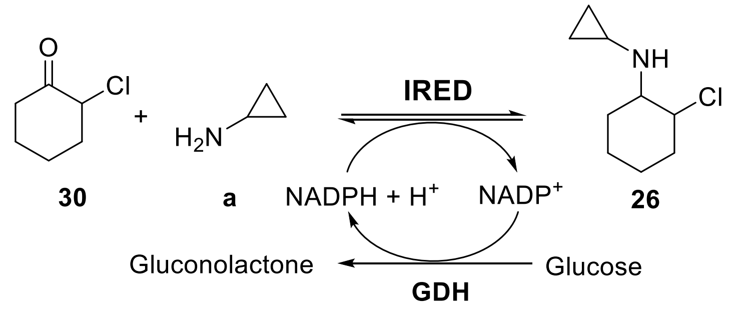

Figure S5. Scheme of reductive amination of 2-chlorocyclohexanone with cyclopropylamine for the synthesis of **26**. Cofactor regeneration system consists of GDH, $NADP^+$ and glucose.

Test reaction **26** chromatograms

a)

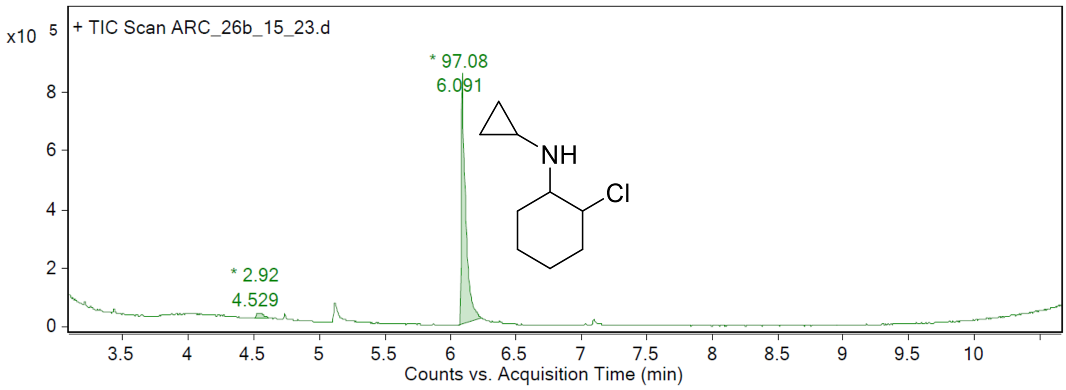

b)

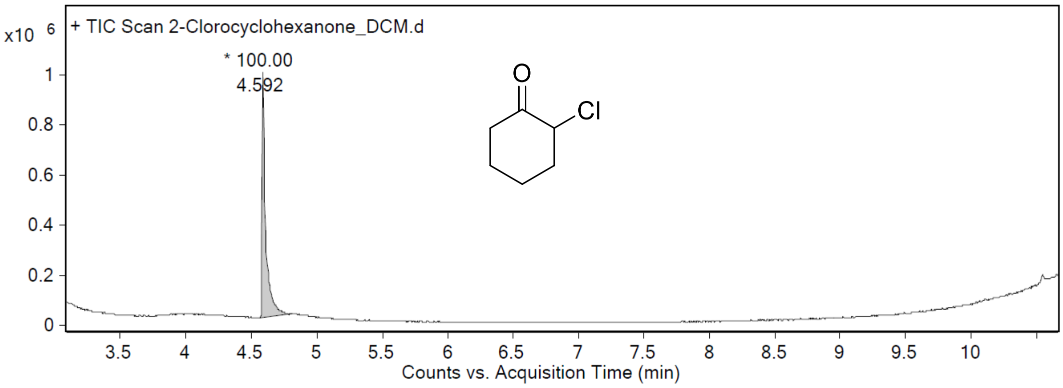

c)

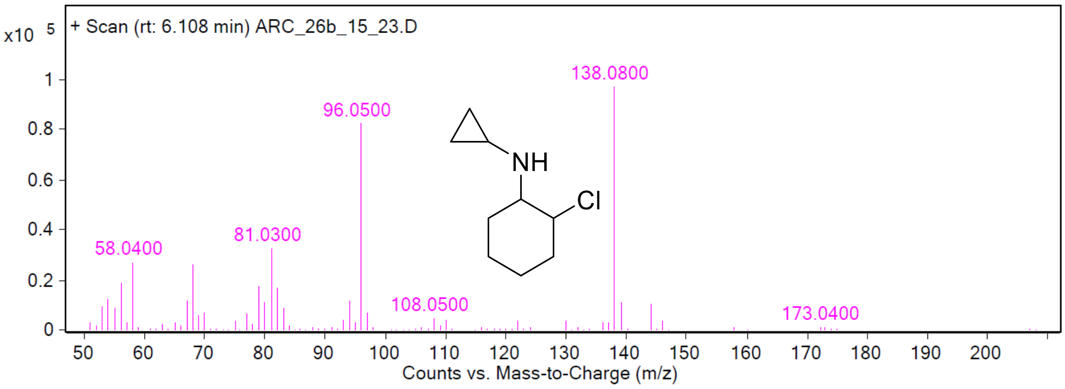

d)

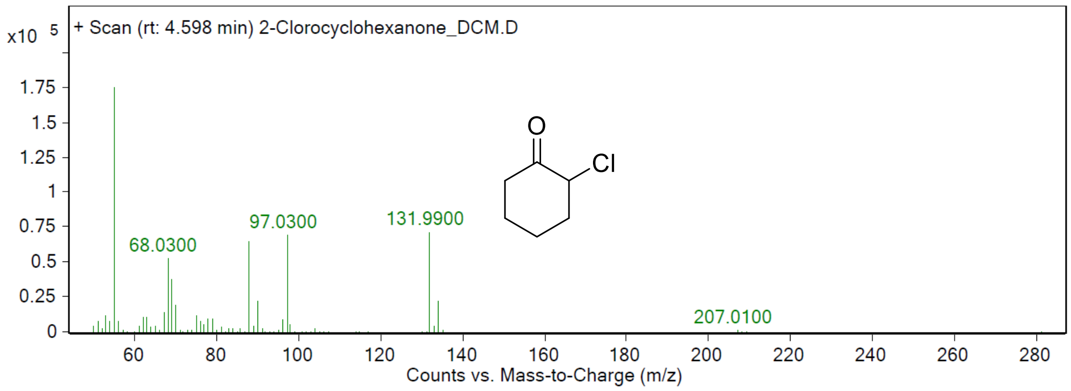

Figure S6. GC-MS spectra from screening for the synthesis of **26**. a) GC-MS chromatogram of 2-chlorocyclohexanone reductive amination with cyclopropylamine which exhibited 97% conversion. b) GC-MS chromatogram of cyclohexanone (**30**) standard. c) GC-MS spectra of peak at R_t = 6.1 min corresponding to GC-MS chromatogram a. d) GC-MS spectra of peak at R_t = 4.6 min corresponding to GC-MS chromatogram b.

Table S4. Retention times from GC-MS analysis for test reaction **26**.

**Compound**	**Retention time (minutes)**
*2-chloro-cyclohexanone (**30**)*	6.1
*2-chloro-N-cyclopropylcyclohexanamine (**26**)*	4.6

Test reaction **27** scheme

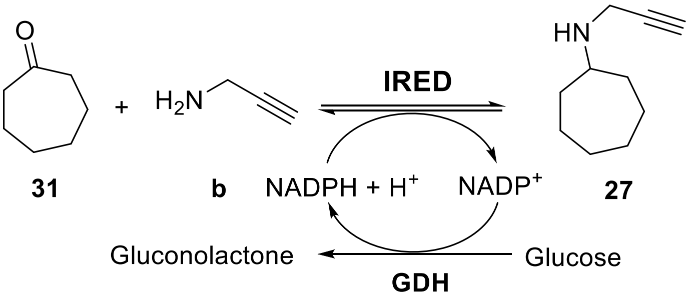

Figure S7. Scheme of reductive amination of cycloheptanone with propargylamine for the synthesis of **27**. Cofactor regeneration system consists of GDH, $NADP^+$ and glucose.

Test reaction **27** chromatograms

a)

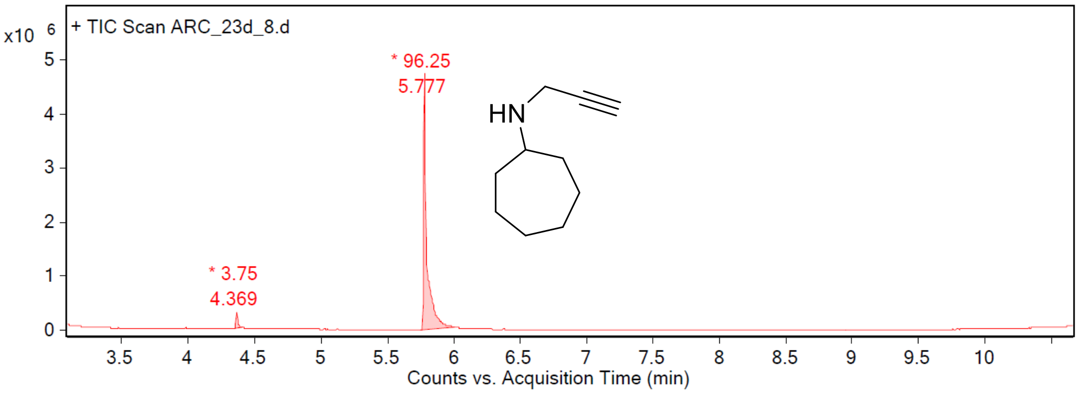

b)

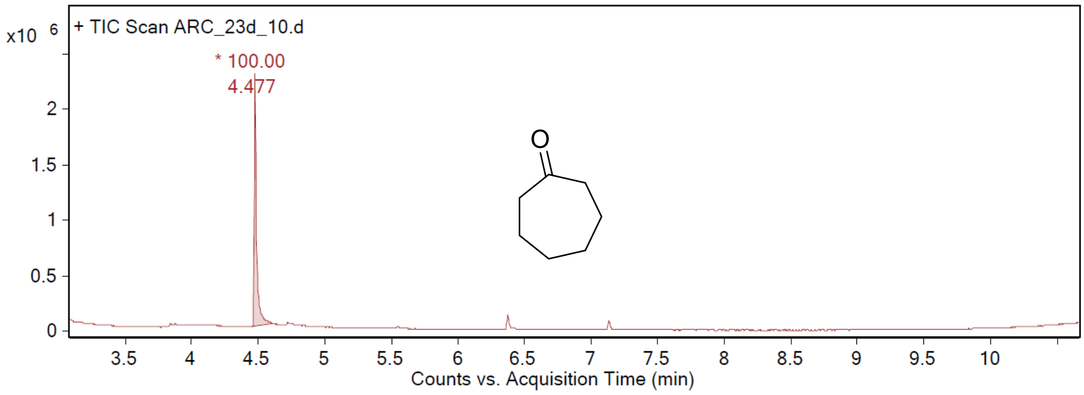

c)

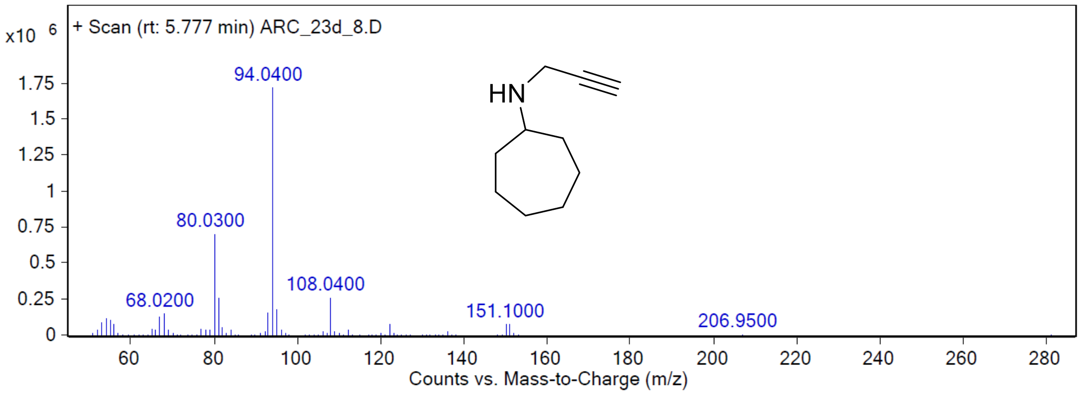

d)

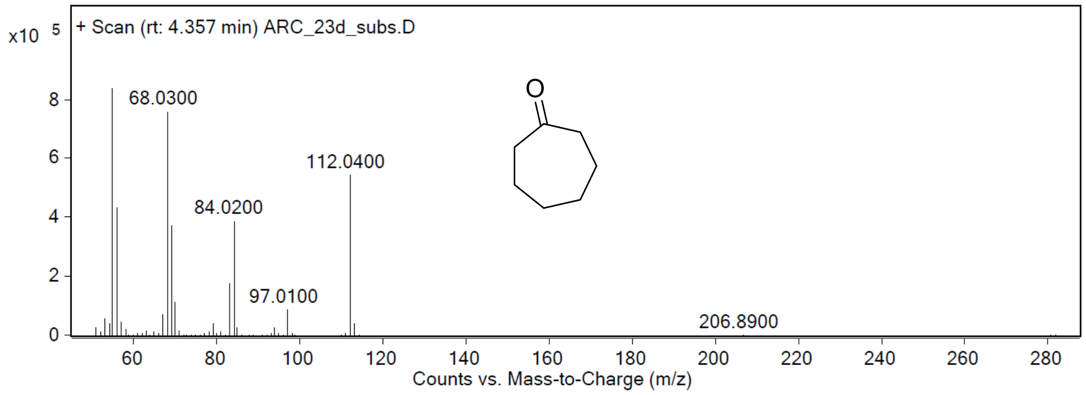

Figure S8. GC-MS spectra from screening for the synthesis of **27**. a) GC-MS chromatogram of cycloheptanone reductive amination with propargylamine which exhibited 96% conversion. b) GC-MS chromatogram of cyclohexanone (**31**) standard. c) GC-MS spectra of peak at R_t = 5.8 min corresponding to GC-MS chromatogram. d) GC-MS spectra of peak at R_t = 4.4 min corresponding to GC-MS chromatogram a.

Table S5. Retention times from GC-MS analysis for test reaction **27**.

**Compound**	**Retention time (minutes)**
Cycloheptanone (31)	5.8
N-propargylcycloheptamine (27)	4.4

Test reaction **25** scheme

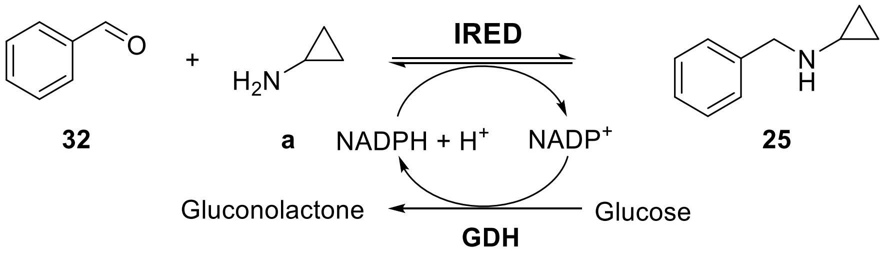

Figure S9. Scheme of reductive amination of benzaldehyde with cyclopropylamine for the synthesis of **25**. Cofactor regeneration system consists of GDH, $NADP^+$ and glucose.

Test reaction **25** chromatograms

a)

b)

c)

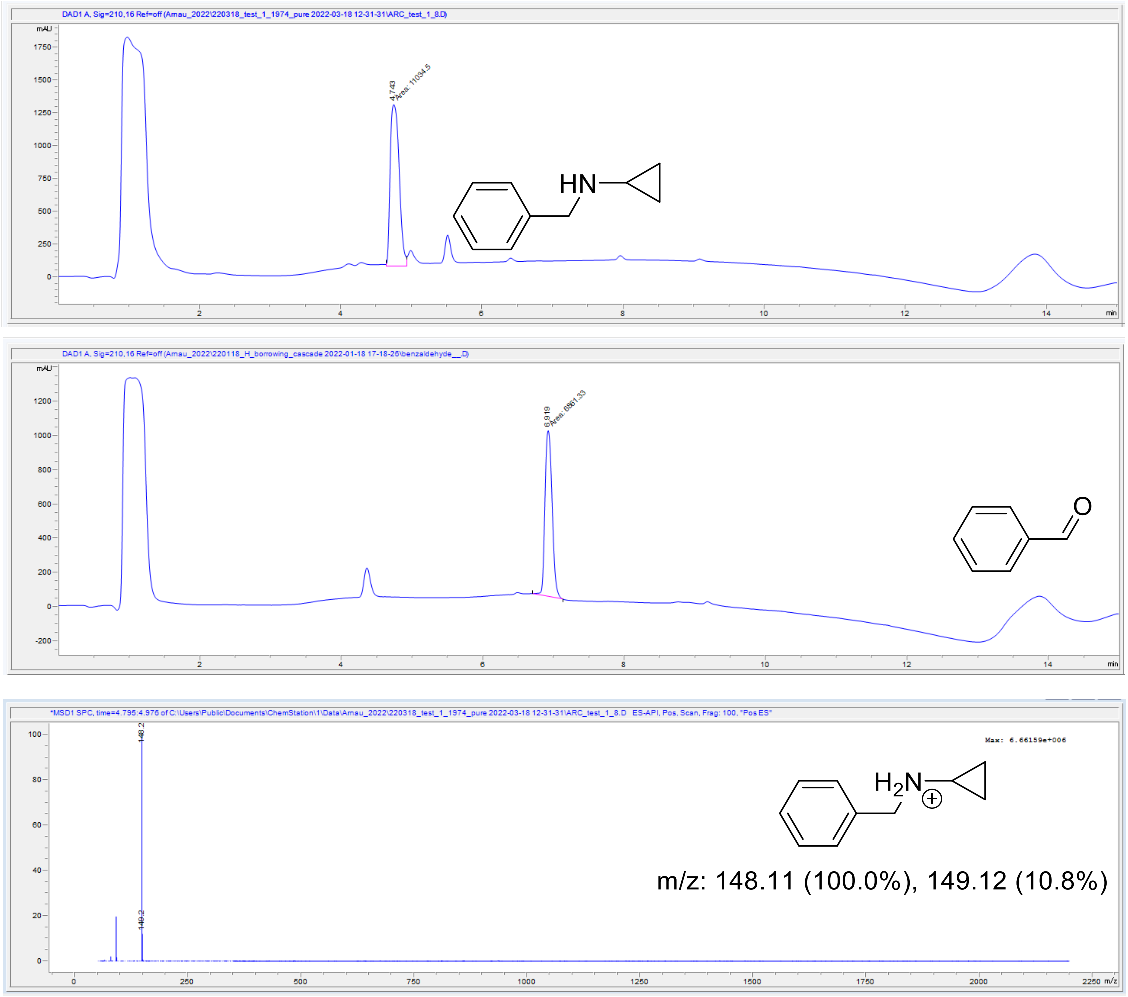

Figure S10. LC/MS chromatograms from screening for the synthesis of **25**. a) LC/MS chromatogram of benzaldehyde reductive amination with cyclopropylamine which exhibited 96% conversion. b) LC/MS chromatogram of benzaldehyde (**32**) standard c) LC/MS spectra of peak at R_t = 4.7 min corresponding to GC-MS chromatogram a.

Table S6. Retention times from HPLC analysis for test reaction **25**.

Compound	Retention time (minutes)
*Benzaldehyde (**32**)*	6.9
*N-benzylcyclopropylamine (**25**)*	4.7

Test reaction **28** scheme

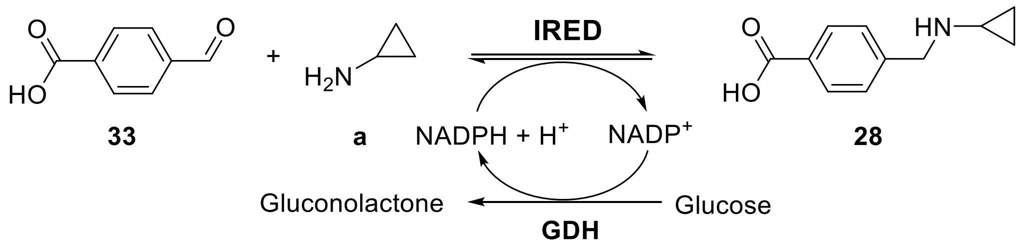

Figure S11. Scheme of reductive amination of 4-formylbenzoic acid with cyclopropylamine for synthesis of **28**. Cofactor regeneration system consists of GDH, $NADP^+$ and glucose.

Test reaction **28** chromatograms

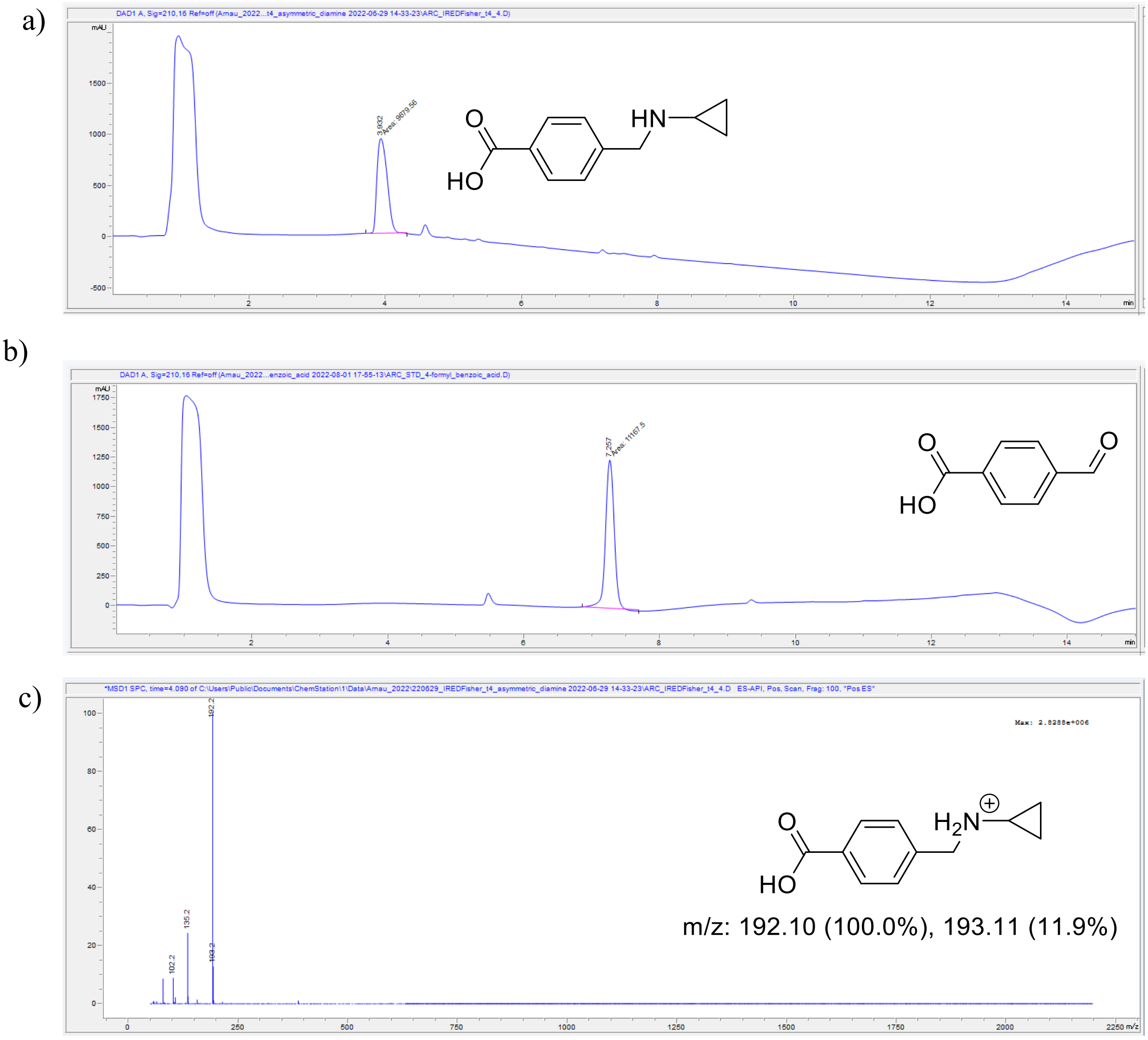

Figure S12. HPLC chromatograms from screening for the synthesis of **28**. a) LC/MS chromatogram of 4-formylbenzoic acid reductive amination with cyclopropylamine which exhibited 95% conversion. b) LC/MS chromatogram of 4-formylbenzoic acid (**33**) standard c) LC/MS spectra of peak at R_t = 3.9 min corresponding to GC-MS chromatogram a.

Table S7. Retention times from HPLC analysis for test reaction **28**.

Compound	Retention time (minutes)
*4-formylbenzoic acid (**33**)*	7.2
*4-((cyclopropylamino)methyl)benzoic acid (**28**)*	3.9

IRED Sequences

>yIRED1

MTNQTAEQVSVTVIGLGAMGRALAGAFLREGHRTTVWNRTAEKADELVARGAVLA
GSVGEAVAASPLVVVCVTDYDAVRELLDPVDLGDRAVVNLTSGTPGAARELAGRV
GAYLDGGIMATPAAVGTAEAVLVCSGSRAAFERYEPVLRGLGAETVYLGEDAGLA
ALYEVGVLGLMWSTLNGFLQGAALARAAGLDAASFAPLAVKSMESVAGWLPDYA
RQIDEGSYPPLDATIDVHLAAMRNLIEESESLGVSTELPRFVKALADRAVAEGRGEEG
YAALVEQFGKPS

>yIRED2

MTEQLTSSSDTDRQAVTVLGLGLMGQALAGAFLAAGHRTTVWNRSAAKAEELAAQ
GATLAATLRDAVAASPLVIVCVTDYPAVRALLDPVSADLVGRVVVNLTSGTSQQAR
ETAEWAAQHGFSYLDGVILAIPSAIATDEAVLLYSGPRAAFDAHEQTLGSLGAATSH
LGADHGLSALYDMAMLSLMWSVLNGFLHGAALLGTAQVDATTLAPFLTQGLHTMS
GWLTATAAQIDAGAYPAEDSTLDVHLAAMAHLVEESEAVGVNAELPRLAQALAGR
AVDEGRGGDDYAALIEQFRKPAPR

>yIRED3

MNAAAPVTVLGLGLMGQALAEAFLREGHATTVWNRTAAKAEPLVAKGARLADSV
ADAVAASPLVIVCVLDYDAVHQLLDPVAGALDGRVLVNLTSGTSEGARETARWAA
EHGAAYLDGAILTDPDGVGTADAVILYSGPHAVFEAHEPTLGLLGGATTHLGDDHG
LSSLHDVAVLGLMWGVLNSFLQGAAVLGAAGVDASTFAPLAAKSVKMVADWVPG
YAEQIDNGVYPAVDATLNTHLASMNHLVHESEFLGVSAEFPKLVRAMAERSVANG
HGAEGYASMIELFRKPSGTRE

>yIRED4

MAHHASTRTSTETSTETSTDVTIIGLGLMGRALADAVLAAGHRVTVWNRTAAKAEG
LVSRGARLAESVESAVLASPVSIVCVADYESLQQAFASVSGELDGRVLVNLTSGDPE
QARELARWADQGGARSLDGAIMAVPSTIGTADAIILLSGSRAAFESHQPLLAALGDA
TFLGDDHGLSALYDVAGLSVMWGVLNAWLQGVALLGTAGVDASTFTPFATQVAQ
ETVGWLSGYADQVDRGSYPPDDATLATHAGGIAHLVRVSEQLGVDAELPRLFQSLV
ARALAAGQADQSYAALIEQLTPGARTS

>yIRED5

MTNNTHKTDSPVTVIGLGLMGQALAPAFLKAGHATTVWNRTAGKADHVVSQGAQ
LAPTVGDAVKAGGPIVVCLTDYAAVRELLGGSEVDLAGAMLVNLTSGDSAQARET

ARWATERGARYLDGAVMAVPPAIGTDAAVILHSGPEADFQEYEPTLAALGTVTHLG
VDHGLASLYDAAGLTMMWSILNAWLQGIALLRTAGVDAATFAPFAQQMAATVAG
WLPYDAEQIDKGDFATEVSTLGTHTRAMEHLIEESETAGINAELPKLIKALADRAIAA
GHADLEYAVLIEEFTKPG

>yIRED6

MTSPTDSDVTLIGLGLMGKALAGALLGAGHRTTVWNRTAAKAEELVAQGALLADS
ADSAVTASPVIITCVSDYASLQQVLTPVADGLGGRVLVNLTSGDSAQAQEMARWTE
KQGGIYLDGAIMAVPPTIGTAAAVILLSGPESAFTTHQSTFDALGTTAYLGEDHGLSA
LYDVAGLSMMWGVLNAWLHGVALLGTAGVNASTYTPFAAQIAQETVGWLAGYAD
QVDSATYPPDDATLDTHAGGMTHLIEESERLGVNAEMPRLFKALVDRAIAAGQGGS
SYPVLVEQFAQPRATSR

>yIRED7

MNNAPATNTAPVTKNAPAANTAPVTDDAPVTVTVIGLGLMGQALATAFLKAGHPT
TVWNRTPAKAEDLAARGAAVAPTVGEAVLASSLIVVCLSDSAAVHDTVGPHRAELA
GRTLVNLTSGTSDEARETAAWATDFAYLDGAIMAIPEVISRPEAFLLFSGAPEAFERH
EETLKTLGNAVFFGEDPGLASLYDVALLGVMWGTLNAFLHGAALLGAAKVDASAF
APFANQWLSSVTGFVSAYAEQIDRGAYPAEDAALETHLATMKYLVRESAAAGIDTD
WPARIQALAERAIEAGHTGAGYAGLIEVFRGQA

>yIRED8

MNTDSSRVATPAGTDPGRSRTRTPVTVLGLGMMGRALAAAFLRDGHPTTVWNRTA
AKAGPLVAEGATLAASPAEAVAAAPLVVVCLSDHASVRAVLDPVGGALDGRTVAN
LSSGTSGEARETAAALARHGAAYLGGAIMAVPETIGTADALLVYSGPRAVFDRHEPV
LRSLGTGTSHLGDEHGLASLNEMAVLSVMWHVLNGFLHGAALLGAAGVDAAAFAP
LAKAGIETTAGWVADYARQIDDGEYPPLDATIDTHLTTMRHLVAESEALGVDAAPP
ALIAAPAERAVAAGRGGDGYAALIEQFRKPSEG

>yIRED9

MAGDDRRTVSVVGLGPMGTALAGAFLENGHPTTVWNRTASKAGDLVARGATHAA
TVSDAVSASTLVVVCVSVNEIVRKLLSSLDDDLSGKAIVNLTSGTPEEARETAVWAA
EHGADYLDGAIMANPRHIGQLETMLLYSGSPVVFEAHEPTLSLLGGATHVGTDPGLA
SLFDAALGGLAWSTWSSFLHGAALVGTEDVKAMAFAPIAAQWLAAVAGIMADYAR
QVDDGHYPGDEETLEINAAAMDHLINASRAQGIGVDLPNFLKALTERAIAAGHGSDS
FASVIEAIRTPSA

>yIRED10

MTKNPRTPVTVLGLGLMGEALAGAFLREGHPATVWNRTAAKADRLAGRGATRAGS
ARDAVAAAPLVLVCLTGYDAVHEVLGPLGDVLDGRVVVNLTSGTSAEARDTAAW
VERQGGTYLDGAIMAIPDAIGTPDAAVLYSGPRAAFDLHERALGSLGGTATYLGAD
HGLSALYDVGVLSLMWNILNGFLQGAALLGAAGVEATAFAPVARNGIETVAGWLA
GYARQIDDGVHPAADSTLDTHLAAMEHLVQESESHGVDAGLPRFIRALAAEAVAAG
RGGEGYTALIDRFRKPSDHA

>yIRED11

MTTIGFLGAGRMGTALVAALCRTGRTVHVWNRTAEKAQALAAFGARPERTPEAAV
AAADIVMVNLLDYVASDALFQRPEVTEALKGRLLVQLTSGSPKTARDTGAWATVK
GIDYLDGAIMATPDFIGGVETQILYSGARDRYEKYEAVFAALGGKSAFVGEDFGVAS
ALDGALLAQLWGTLFGTLQATTIARAEGIALDDYAASLKLFRPVIDGAEVDLLSRVR
DGRDRGDEATLATIAAHNTAFQHLRHIVAERGLNPAITDAFDSYFAAAIANGHGNDD
FAMLASYMVSQ

>yIRED12

MKTVAVIGLGQMGTTLARLFIEAGMQVRVWNRTRSKAEPLASRGAIVAATAAAAM
ADAEAVVICVHDYRATHDILSDVAVKSALKGKLLLQLTTGSPQDARDMAELAARIG
AGYLDGALQVAPEQMGQPDTTVLVSGSGEDHALARELLAVLGGNVVYLGEDVAA
AATMDLATLSYVYGASMGFFQGAALAQAEGLDVGVYGGIVEAMSPSFGAFLRHEG
NVIDNGDYAVSQSPLSISIDATGRIEQAMRQKGLRSELPSLIARLLRDAEEAGYGNEE
FAAVAKILRGAAEPAPVR

>yIRED13

MSDGKKSAPEVAVIGTGAIGSALVHRLLGSGYDITVWNRTESRATELVAAGALPARS
VREAVSSSTLVFLTLKDYTAVEQCLTQLDMDLSGHTIVGMYTGTSSEARLAAQRAT
TLGAHYIDAGIQASPEMIGTDTATILYSGSRHAFEQHKTTLQLLSKPRFVGETPEAAA
VWDLALFGVWYDAQLGLLRALGAAHEAGIDVVEFSHTASTQLGHVVTGVSATVSE
MQQATYPPGPADLTEHLAVVRHLIELRSGRRLGNGGLPEVAARIEALIADGRGDEGL
TATVG

>yIRED14

MTGTGKITVIGLGAMGAAIAGALLAKGFHVTVWNRTPEKAALLVQRGAVLASEPAA
AIVASPVTIVCVSDYDTATKILDMTGVPATLKGRTLILLSAGTPNDARSLDAWVHAE
EGHCLHGGIAAWPRQIGTAEAMITASGDKAVFDQQRSVLEALAGTVHFAGEGPGTA
ATLTTAAMAYLAGNWIGFCYGALICEKEGLKVDEFGQLMEGFTPMLAAEVRHMGE
VIQHNRFSDPESTINTTGLDLRLLLQHTREAGLHTALPELAADIFNKAIAAGYGKEEH
AAIIKILRKQDS

>yIRED15

MRTVVIGTNPRAAAIAAALAVPATDTVAGHADLVVVCVEDYSLVPRLPGDADIVNL
TSGTSEDAARTADLFPGRYLDGALMAHPEHVGRPETVIVYSGSPDVFARTEKQLARL
GSATYLGPAAGTASLYDAAMLNFAWATLTGYLQTAALLTSEGVAARAFTPMLNEW
MRTTVAAVIDDYADQIDTGQYPGDEEWLELDEPLMRDLITVTEQRGMDAALPKVV
QDLTARGIAAGHGAESFASLVEVIRGGGPGAEPATA

>yIRED16

MTRKNVTVIGLGAMGSALANALLAKDLKVTVWNRNKAKAALLAHKGALVAENVK
AAVEASAFVVICVSDYKAAAKIFSEAGKELLHGRTLVQLSTGTPKEARDFDAWARQ
QGANCLNGDILAWPRQIGTADATITFSGNNEIFKQFETELNALAGVISYLGEEPGASA
VLFSAVLAYLAGNWIGFCHGALICEKEGMRPDAFGELIYNISPILATESKHMGHVIQH
NKFTDPESTINTTGLDLHLLVQQSKEAGISDELPKFAAHIFQRAIDAGYGGEEHAAIIK
VMRGAG

>yIRED17

MRNTRATQRPSRSGDRKHRDRCCGCWRGDRHPLHGPHKENRDATHAPHTHRTPRP
ARPRAPHRHGRRLVSAPPVAVLGTGRMGGAMARALIGAGHPTTVWNRTPERTRPL
VAAGAVTADTPAAAAAAAPLVILCVTDDDAARAVLTDAAAALQGRTLVNVTTSTP
EQARAAAALATSYGADYLAAPAMADYRTIGRPETHIMYSGQREAYDRHEDTLHAL
GKAARYVGADPGIASLFELAMLSLFFEVWTGYLHVLALIRHEGVPAGDFAPVAART
FGEISHLLPLIAQQVDSGDHDPAAYGGVAEQAAFTDTVIELRRTRGIDTTRLEHLKAL
MERAITEGKGEQGMSALFETLLAAPPGT

>yIRED18

MPDETNSTSSADSTTAADPTVAPTPVTVLGLGAMGQAIAGAFLKAGHPTTVWNRSA
GKGEDLVARGATRAATAADAVRAGELVVVCVVDYDASQAILEPLAADLSGRVLVN
VTSDSPERARAAAKWAAGHGIAYLDGAVMIPTVMVGTPEALLFYSGDEAAYKKHE
ALLKSLGGQSAYVGADHGLAAVYDLSMLDFFWTSMAGLVHGYALAAKDGVPASA
LAPFLQSHISLLAVLVGETAKDVDAGVYPGEEGNLAMELEGVEHILHAAELRGLDVS
VLRGVRDVAKRAVDLGHGADSWTATVEGVRNPGA

>yIRED19

MAKVSVLGLGNMGAAIAWAFHKANHDLVVWNRSSERSRPFADADITVAENPVDAI
AASSFIVVCVDSNPVADSFLQADDVAAVLGGRTVVQFSTSTPKEAEASATWLASRSA
HYIDGAIHCSPGHIGTDDAAFLLSGDKNIYGAVRPFLACLGGDQTFIGETVRAASALE
LASVCEVYGRFAAVSHAALICEAEGIPLDQLAALMPEGGFSQRYVETIRDGAFENPG
ATLRIWDSAIQRVRAQGHDAGIDTRFPDYLADLFNEAINDGLADEHVMALIKVLRKA

>yIRED20

MRQAPANTARPAPVSVLGLGAMGGQLARALLAAGHPTTVWNRTPAKADALVAQG
ARRARTVADAVAASPLAIVCVLNGEVVHRILEPVGAALSGKALVNLTSDTPERNRR
AAAWAAEHGIAYLGGAMLMPPTLVGRPEATILYSGSRRAHAEHEATLKALAGRAPY
LGPDAGTATAYDVALLSLYYAGLAGMVNAFALAGGEGIPAQDLAPHLDVILGQFPS
VAVSMARDIDAGRYSGEADVLAIHTAGLAHIIETSRSHGINTDVLRAVKGLVDRAIA
AGYGDAEFAGVIEEMRA

>yIRED21

MGSAFVRALLRAGHKVTAWNRTTARAEALAPLGADVLTSAGEALVSAQLAIICVGS
TDDVRHLLDAADPTQLRGQALLNVTSGTPADARSLRDYAHERGIHYLDASIGAYPE
QIGAHDARILVAGDEELWQAHSAVIRDLAGASLLVGTDYAAANAIDAALTGAFYITS
LTAFMEAVRFMGSFGISHEVLTSLSTYSISVLDHQAKLALDRVASGDYATDEATLKV
YADAAAAFAAGLGETGEAPMVETTARVLREAVAAGLGAVDIAAIAAPRD

>yIRED22

MNSPTRDLHPRTTRPLSDRTTSSVRRVAVLGLGAMGIALARALAAAGHEVTAWNRS
PKDPVALGFDTVGIRVAAAVEDAVAEAEAVLVCVRDHHASRDVLATIASQIEGDVP
VVNLSSGTSDQAVASAAAAADLAYVTGAIMVPTPLVGTEDNLVLVAGPAGAVRSA
VPVLEGLGGTIDVLGEDHALPPVLDMAMLDMYFAGMYAFLHSAALVRRHGIEPTA

YLPYARGLHATLGVELDGLAASFENGTYDGGQASLEMCLSFLEHIVATSEDAGVDP
ALPLLVRDATRRALATSTGDTDWDVVAEGLAHPA

>yIRED23

MGRHDEVARRSEAGDGPIGAGGARVSVLGLGLMGAAFARALVDAGHSVTVWNRS
PGRCAEFVARGARAASTPEDAVRSSDVSLLILSNYEVTADLMTQVGDAVSGRTLVN
LSTGDPGSAVRLFDRMTSHGAAYLDGAIEAYPSEIGRPGTLISYSGSATAWSTYRELL
LTLAGRSHFLGDDPGAANVLDAAMAGAFYNSALGAFHEAVSFATSSGIALADISFSI
DYWIDLLRTHLYESVDEIGSGDYTTDQAALNTYLAAVRSWRTTVMAEGQRAHIMT
ANMHNLEAACEAGLGSLSLAAHHLTMK

>yIRED24

MDVRISMIGLGRMGSALARGFLRCGAQVTVWNRDAAKAEALRGEGATVARSLAEA
LKASDLIAVSVLDTAASHALLFSPASAPLLRGKAVLDFTTSTPAVARAAMQHATDAG
AQYLDGALMCYPSGVGTPNATILFSGAKTVFDRYEPQLKALGNAMFLGEKAGAAA
TLDLALLDFYYGATAAMLHGVAMCQSEGFPLEIYAKALGGFAPIVAQTAELAATMV
PARNYTGSEATNDIHAAALALIVQAAKDAKLDPFPEQIMGYFKSALAKGHGPDEIVA
LVETMRP

>yIRED25

MGNRDTGLTPVTVLGLGLMGQALAGAFLRDGYPTTVWNRTAAKADGLVAEGAKL
AGSVRDAVAGGRLVVICVSDYEAVRELLDPLEDVLDGRVLVNLTSGTSAQAREIGE
WAARRGGGYLDGAIMAVPAGIGTADATIVYSGPRTDFDLHEPVLRSLAAGTDRATG
TDGTAGAEAAGVGAAGTVYLGGDHGLSSLFEVAVLSLMWNILNGFLHGAALLETA
GIKATAFASLAEQAIGTVAGWLPGYAQQIDDGAYPALDSTIDTHLGAMEHLVQESVS
LGVNAEFPKLIKALTDRAVAGGRGGDGYAALIEQFRKPSEARP

>yIRED26

MREMSEVTVIGLGLMGQALAQAFLAKGHATTVWNRSAGKAEQLAAQGAVRADSA
RAAAGASPLVVVCVSDHQAVRELLDPLEDVLAGRVLVNLTSGTSEQARETARWAE
RRGIDYLDGVIMDVPQGIGTAGAVLYYSGPRPAFDRHEPTLRSLGEGTVYLGADHGL
SSLYDMAMLTMMWSILNGFLHGAALLGTADVPAATFAPAVVKGMSTVAEWLPGY
GAQIDSGKYPALDSTVDTHLAAMEHLVHESEALGVSAELPRFVKALADRAVAGGH
GGEGYAAMIELFRQPAAAGG

>yIRED27

MSSHPLHKGRTKHDHDTSPVPVTVLGLGMMGRALASAFLRQGHPTTVWNRTAAKA
RDLTAQGARPAASVGEAVAASPVVVVCVSDHEAVHALLDPLDGALEGRVLVNLTSS
TSAQARLTAAWARRGGADYLDGAIMAVPDAIGTAEAVIVHSGSREAFERHEPALSSL
TANTSYLGEDPGLAALHETAVLSLMWSVLNGFLQGAALLGAAGVDASAFAPLASR
GIATVADWLPGYAEQVDDGAYPVLDSTIDTHLATMEHVVHESEALGVSAELPRFVR
DLTRRAVADGHGGEGYAAMIELFRAPSGIRS

>yIRED28

MNTNTKTHEPRAPITVIGLGLMGAALARTFLKAGHPTTVWNRSAKKADDLVAKGA
TRAAILSEAIRASPLVVLCVLDHDAVREVLQNAGDAFTGRVFVNLTSETPAQARETA

AWASARGIDYLDGAIMDIPQGVGRPQATLLYSGSRSAFTDWQPALSCLGTGTYLGT
DAGLAALYDLALLGIMWSTLAGFFHAAALVGTEKIPATTFVPMATGWLSAVAGFLP
RMAEQVDSGDYETDVSTLGISAVGLGHLVHASEEQGLTSDVPGSLKALFDRAVAKG
HGGHALASLVEVIRRSKPQ

>yIRED29

MGDNHTSVSVIGLGLMGQALAAAFLKAGHATTVWNRSADKADGVVANGAVLAAA
PADAVAASDLVVVCVSTYDVVHDVIGSLGDALRGKTVVNLTSGSSEQARQTAEWA
EKNGARYLDGAIMITPPGIGTETAVLFYAGDQAVFDAHEPVLKLLGAGTTYLGTDHG
KPALFDVSLLGLMWGALNSFLHGVAIVETGGVKAQDFLPWAHMWLDAIKMFTADY
AAQIDAGDEKFPANDATLETHLGALKHLVEESEALGVDTELPKYSEALMEGIIAQGH
AKNSYASVVKAYRRPAR

>yIRED30

MTHGVNNPVTIVGLNARGAALARALAVHTRVTVWDGAGHRSEAPHIEGVSRAGSA
AEAFTASPLAVLCGDDYDHVHRVLETAEPHVSSVDVVNLTSGTGDQAEQVAAWLG
ARGVDYLDGALMAHPEHVGDPDTVLVLSGSRPVFDRHAAVLARLGSPTYLGSDPG
AAALYDVAMLNFAWSTLLGFLETAALLGTAGVSATSVSPLLTRWLSGTVAEVITGY
ASQVDDRRYPGDQDWLELDAPLMDHLVEAARARGIDTALPELIRSLTAKGIEAGHG
RASFASLVEVLRV

>yIRED31

MTTAPDPATPVTLIGLGPMGRAMTRSLLAAGHPVTVWNRTASRADGVVADGAVRA
ASVAAAVRASPLVVLSLTDYAAMDDVLGDLGTELRDRTIVNLSSEGPARTREAADE
MRAHGARFVVGGVMVPPPMVGTAAASVFYSGDEAAFAQYADVLGHIGEPRYLGTD
PARAQLMYQAHLDVFLTGLSGLAHATALLGRAGFPASEVMPGLLGMLAATPDMVA
DGEPLWAQIDRREYAVELSSATMMGASADHIVLASEELGVDLTLPRAVQGHYRRAI
EDGHGAEGWASIVEGIRAPH

>yIRED32

MASICVVGAGRMGSALARAFLRAGYVTHVWNRTPAKGEALAALGARFVPSLHQAI
AASDIVVVNVIDYAAADAHLRSASVTRALGRKLLVQLTSGSPSQARQTGEWAKGHG
VGYLDGAIMATPNFIGEPSATILYSGSQHAFDENRDVFLALGGNAVHVGDDFGHASA
LDIALLSQLWGTLFGTLQAIAVSQAEGIELDAYARYLQPFKPTIDGAVADLVTRARD
GRYRGDDQTLAAIAAHYSAFQPLLEVSRERGLNRAVPDAFDSIFKAAIAAGHLQDDF
AALTRFMR

>yIRED33

MSNPNAAQAPVTVIGLGLMGQALAAAFIKQGHPTTVWNRTPGKAEQLTADGAILAQ
SAGKAISASPLVIVCVSDYDGVHEILGPLQGALAGKVLVNLSTGTSAQARETAEWAA
QRGARYLDGAIMAIPPVIGTDGAVLLYSGPQDSFEEHAATLRALGTPGTTYLGGDHG
LSALHDMALLGLMWGILNGFLHGAALLGTAGVKATTFAPLANQMIKEVTEYVTAY
APQVDEGKYPATDATLTVHQDALEHLADESKAVGINSELPRFFKALVDRAAADGRA
DESYAALIEVFRKEAAA

>yIRED34

MTTDTHADTHIDTAPAPAPVAVLGLGLMGHALAAALLRAGHPTTVWNRTAAKADG
LVAAGAVRAETPAAAVAAAPLVIACVTDYDALHAFLDPVDPAALGGRTLVNLTSGT
SAQARDTAAWAAGRGLTYLDGAIMATPQAIGTAETAIALSGPRDAYDRHTPALRSL
AGDTPYLGADQGLTSLYEMAMVGVMWSLLNGWLQGAALVGTAGVSAAEFTAVA
AKGLPAVLDWLPGYAKQIDTGTFPPDDATLDTHRVSMAHLVQESESLGVNAEFPALI
KSLADRAAAAGHGGEGYPVMIEEFRRPAARPA

>yIRED35

MTTNSSQVARMARKHDGSPVTVLGLGLMGQALAGAFLRKGHATTVWNRTASKAE
RLVAQGAKLAGSVRDAAAASPLVVICVSDHEVVGGLLEQLDDVLDGKVVVNLSSG
TSAQARDLAARAARRGGALLDGAIMAVPDEVGSKDVAVVYSGSRAAFDAYEPVLR
DLAEATYLGEDHGLSSLYEVAVMPLMWGILNGFLHGAAMLGAVNVNASAFAPIAE
QAIGSATRWLAGYARQIDEGVFPPVDVSIDLHRAEMQHVVAESEALGVNAELPKFIR
AMADRAVAAGHGGESYPAMIELFRAPG

>yIRED36

MSVFHFPPIALSALANPQSSSGSRFIFDPYLNVTAYPKVRNLPAAGRQPYQRASQILEL
DLMTTIGFLGAGRMGTALVRTLLKNGHHVHVWNRTAEKAEALAAFGAVPASRPEA
SIDGADIAIVNLLDYSASDAQLRKPAVMQALKGKLLVQLTSGSPKTARNTGIWATDN
GIAYLDGAIMATPNVIGSSETLILYSGSASHYEKHATLFAELGGKSAFVGEDFGKASA
LDSALLAQMWGTLFGTLQALAINRAESIDADTYAGFLPLVQPVIDAAEQDLMQRVR
DGRDRGDDDTLASIAAHGVAFHHLREIVRERGINSAISDAFGSLLETAIAKGHQDDDF
AILARFMMAE

>yIRED37

MMHTGTPVAVLGLGLMGSALAGAFLRAGHQTTVWNRTTTKADDLVARGATLAES
PAAAIAAAPLVVVCVSDYDAVTAILDGALDGRVVVNLTSGTPSRARALAEEVVRRG
GSYLDGGVMAVPEAIGTADAVLAYSGPRPLYDAHESTLRGLGTGLHLGDDPGLAAT
HEMATLVLMWGMLDGFLNGAAIVGTAGVSATAYLSIARRAIDTVAGWLPDYARQA
DNGSYPADDGTIDTHLAAMAHVVEESESLGVNTELPRLVKALAERAANAGHGGSG
YVALMEQFRKP

>yIRED38

MGAKKKPVTIVGLGPMGRAMTHAYLDRGHQVTVWNRTAGRADEVVAAGAVRAD
GVADALAAGELVILSLTDYDAMYAILEPASAALSGRVLVNLSSDTPGRAREAAEWA
AGHGARFLAGGVLTPPSGIGQPTAATFYSGPEEVFTAYEDTLKVLTGTDYRGADPGL
APLHYQIHMDMFWTTMASWLHALALAGAHGISAEEVMPYAADATSIQQFLAFYTP
RIDAGAHPGDVDRLTMGTASVEHVLHTTRDAGVDTALPEALLGVFRRGVAAGHGD
DSFTSLLEVFREREAA

>yIRED39

MTATTKHPAISVIGLGSMGAALARALVSKGFQVTVWNRNMEKAQPLIAADAIAAAD
AKAAIEASPVIVVCVSEYKVTRKILEADGVAPALKGRTLVQLSTGTPKDARELDTWA
KQQGACCLNGDIMAWPKQMGTDAATISVSGDADVYRQQEDVLRALAGNVVYLGA
EPGASGGLFHAVLAYLAGSWIGFCHGALVAEKEGLRPEDLGILLEQISPILSAELKHM

GEVIQHGRFSDPESTVKTTGEDLLLLVQQAKEAGINSELPEFAAKLFKQAMDAGYGQ
EEHAAVIKVLRQTA

>yIRED40

MSDVASSPPPVSVLGLGSLGSALARALLTAGQPTTVWNRTAGRADDLVTIGASQAPT
AAAAIAATEIVVIAVADEAAVRGVLGSAAGELAGRVLVNLTSGTPEQARSLAEWAT
AQGAAYLDGAAMSGVRLVGRADALFLYSGSAEAFTAVEGTVSAFGSAAHLGADAG
IASLYDTALLGVNMSLLTGFYHALALVGSAGVDAREFAVVATGYLPFALGLLADHA
RQTVQGQYPPDEGTLDVLASAVDHLVTTSARAGVRTDVPDGIRALVQRGIDEGHGE
HGPASLVEVLG

>yIRED41

MIESTGSATAVSILGTGAIGGKIAQTMLAAGTTVTVWNRTPERTDALAARGSARAPS
AAEAVAASPLVLVCVTDYAAVDAVLEATADTIAGRTIVALTTGSPAEAHQAAHKAE
QAGAAYLDGGVQTEPENIGSNAATFLYSGPRSAFEEHRATLETLGSAQYLGADPAA
AAVQDLTLFGLWYDAQVAYLRALETIRSTGIDVEDFAPLAATQLGHVVHATAGTAH
EVKTRSFPRGPADLTEHGPVLEQLIELRRGQHLGNGALDHIHHLVQQRIELGHGREG
LTTIIE

>yIRED42

MSQRVAVLGAGSLGAVIARAFAAAGHPTTVWNRTPDRLSALVADTPALRPALSVPE
AIADADLITLVVADAAAARAVLDSAGDAIAGKIVVNFTSTTPEQTQVLAGRVGAGYL
DGAAMSGTRLVGDPTGLFLYAGDATTFADAEPTLRALGIARAVGTDPGDASLWDTA
LLGLNLGVLGAFYQAVALIGGSAEKVAAISAEYLPFATGLLADHARQIDAKQYPDD
DGSLAVYAAAVDHLIATAADRGVGTDVAVPVRSVINRAIEAGHGDDGLASVTEVLR

>yIRED43

MTTNTSNPTAPGLTLLGLGQMGAALAGRMLDQGRAVTVWNRTPERAAPLVARGA
ASAETPAEAIARNGLVVTCLLRYASVRETLDPVAASLRGRTLIDLTTTTPDEARELGR
WAAEHDVEYLNGAIMATPYQIGTPVARILYSGAPDVFARHRDLLAPWAEGVYDGPD
AGAASLIDLAMLSGMYHMFAGLFHGAAMAATVGMSAQDFASRTIPLIESLVAHFAA
DAAIIDGGDYSVPGQQSLDFSDLSDILRTSEEAGVDSTTIAAIQALIHRQREAGHGAEG
FARIYETLRHPESGRDEATEAVRGSA

>yIRED44

MGAALAARFLVSGVATTVWNRTASKAAPLSEAGAHVALDPEEALWRSAVCVVCVE
DYSQVRDLLSRGVGRGENFHGRTIVNLTWGTVPEARDARDWVEMYGGRYLDGAIL
DYPANMGPNALVLYSGDKAAFDEHAELLSTIARPRFEGTDPAAANALGMAGALFHN
IALAGFYEAAAYAAQHGIAARTLFDLTAEIGFDLCARAFSDGLNRIESDDYHTDQSTI
GIHFDTTLVQEAGFAEIGQAGTLNKAVRDILESATSAGEGGLAMSALYRRFLQIH

>yIRED45

MSLAIIGLGNMGSAIAEAAITGGHQVAVWNRTPEKAEAFADRATVAESLAEAIAATD
MQVWVLSDYETTEAVLAETGVSAALAGKTFVQLCSGTPAEARALSAKVTDLGARY

LDGKIFTYPARVGAEMTRFAYSGDEVVYKACAPTLKTFGARSGWLGPDPGFAAAAD
LAWLSYLYGSMVGLFQGLAFTRAEGMPDEAVFGSVPSWLVEIDAEARYSKELIERG
DFHGGQAELDVHFAAMQHLLDTAQATSISNAFPRMIVDVFGEALERGYGGMEITGG
LEVFCKP

>yIRED46

MSDEINVEPTAAAVIGLGQMGTRLAQAFLAGGYSTTVWNRSPAKAEALAAQGATR
AANAGEAVAASSLVVVSLPDYATVRTVLDPVAGQLRGRTLVNLTSGTPHDATAAA
AWAAGHGAAYVDGAAMSGTRLVGRPEALFVLSGSRGAFERHRTVLRSLGNSTYLG
ADPALASVYDTALFGLAWGALAGFYHSLALVRSAGVDPAEFGAVATAHMPFVTSL
MTDHARQADEGVFPDDDGTVDVHLAAIEHLSDTSDTQGIGTQVPALFRSLLRQTAA
EGHGASGIASVIGAIAKGGRDA

>yIRED47

MSATNVTVLGLGAMGRAAAAVLAGGGVPVTAWNRTVRPGVPDGVRVAPSVVEAV
ADAPLVLVSVTDQAAATEVLAAAGNAVRGRVVVNLTTGTPAETAALAADLAGRGA
TYVGGVVQAQPEQLGTAAAMLLVAGDAEALERHRPTLDLLGAVVRVGDDPERAGR
YDLTLMGLWYDVQLAVFAAFELAGGDPETFVPFARRQLGFVVDGLPGVARELRVG
AYPRGPATLVEHARVLAQLVELRAAHGLDTSALERADAAARRLIEAGHGEDGFTRL
AAS

>yIRED48

MDKKINLSKQVTVLGLGAMGTALAEAFIKAGYTTTVWNRSADKTVPLVKKGAVAT
STVSEAVSASELTVICVLNYDVLEQILKPVAGELRGHTLVSLTNDTPKRARQMALWA
EEQGIDYLDGSVLVPVTEVGKPEALLLYSGPRNIFDKYFPVLKALGGKSTYLGEAPG
RAALYDIAMMNIFFTSLTGVLHSYALLNAEGEQAEEFLPYAREIFGVAEHFLENFALS
ADKAHYPGHLDNITMEAAGVKHVVEAGRDAGIHTGVPQAVKDVFDRAIEAGFGAS
GMPSAIEGIRKAVRAETR

>yIRED49

MSAPDTPDDTTSNQTPRVAVLGTGLMGSAIARSLLNNDYPVSVWNRTPGRCDPLRE
LGARTVTTSTEAIRDNDVIILMVLDYTAARTLLMDAADHLQGKTIANLVTGSAAEAE
DLSAWVSGQGADYLDGIIAAYPGDIGKPSTLFYYAGNVAAWSRYKTLLTALAGAAT
YVGSKPSAANILDAAMTATFHTVSIGAFLEGMSYARSAGVDLSEIKRTLPYWLDLLS
QEINVALDSIQADSHTTDQATLETYLVALRTINKAMVGDGERANLLGAAVDNLERA
HAAGHGQEALSAQILTAKANT

>yIRED50

MKHSKATFIGLGQMGAALVPPFIDAGLQVTVWNRSASKADPLVALGAKAAADFVA
AVSASDLIIVCLADYTTSNALFHQDEITPLLEGKTIVQLTTGDGREAELGALWAAEVG
VNYLDGAIMDYPTKVGGPECLLLVSGDRAVWDSCETQMKLLGGRMTYVGSKPSSA
NLLDGSLLTMYYGNTFALMQSAAMLEAEQVDIKDFKTALNAFKPVIESTWKRTIEAI
DQQDYSGNEASIHVHSLGVQSLLKRAKKSGVEHGLLQLFSEYVDQTAARGHEADEL
PAAFEVFRRKG

>yIRED51

MAEGDLEIGMNEISMIGLGAMGTALAMAQIEAGRNVTIWNRSSQKMAPLVALGAK
GAENVAAAIKASPVIIICIDNYAATNAIMNVKDVVEHLPGRCVIQLGTGTPEEARTSE
TWFNAREVDYLDGIIQPYPDGIGEPDAQLLFSGSKSVFQKSLPFLKCLGGDLRFVGEK
VGAAAALDLAGLSCSLGQYVGFAHGARLCEAEGVSIEHFANLYPEGNRLRELAEIIH
HEAFTLRSLHDGASIRIWEECIQRLQAQAQDVNMNRDFPDNISRLFKRAIAAGIGEQD
LAALVKVLRD

>yIRED52

MKNPSPIPLPRVVILGAGLMGSAIARELLRLGCSVDVWNRTPAKTTPLVQEGAGTGT
LQSVMTGPALVLLCLSNYETARAVLAEAEEPLRDAALVNLVTGSPTEAEEFSSWASE
RGARYLDGAIEAYPNDIGQSTTLVNYSGHVDVWTDHRELLLAIAGASTYVGERPGA
ANVLDAAMAGSFYNVSIGAFLEAQSYALSQGVTPQQLRSGLPYWLELLRRSLEEGIS
AVEGGVYDTDQATLVVYHDAVRSWLSTVNHADQRSTLLTANEESLRSAVAAGHGP
RSIFAQHLLTSKSVFS

>yIRED53

MMQPHVPSDTTVTVLGLGLMGRALAHAFLRSGHPTTVWNRTPAKAEQLIADGAVP
AESAAEAVAASPLVVVCVTDGDAVRDVLGSVDARVLEGRVVVNLTSSTSEEARQIA
EWATGSRATYLAGAISASPSDIGGEGAAILYAGPRWAFDQHAGILEPLAAGTTYLGE
DHGLASLYDAATLGLMWGILNAYLHGAALLEAAGVDAVTFAPLASAGVRTVVDW
LPGIAEQVDTGRYPAVDASLDIHVAAMGHLLDENESLGIDTGLPRFVKDLADRAVA
AGHGGSDYAAMIEQFRKPAGHFE

>yIRED54

MTQNRDADRTPVTVIGLGLMGQALAGAFLRAAHPTTVWNRSAAKAEPLIAQGATL
ADSIADAVAASPLVVVCLSDYDTAHKLLDPVGELLDGRTLVNLTSGTSAQAHETAE
WAARHGSAYLDGVILAVPQGIGTADAIIVYSGPRAAFDLYEPVLRSLTEATTYLGGD
HALSALHDMAVLGMMWGVLNGFLQGAALLGAAGVDPAGFVPLVRQGIGIVTDWL
PGYARQIAAGTFPALDATLDTHRAAMEHLAQESESLGVNADLPRFIQALADRAVAD
GRGGDGYAAMIEQFRKPSGARS

>yIRED55

MGRALAGALLAAGHDVTVWNRTPGRDGALLAQGARPAESAAEAVATATTTATAT
AASAASSAAAAGSPLVLLCVRDQSAARAVLDAAGAALRGRTVVNATSITPEDARAN
AADARARGFRLLEAAILTPTPTIGRPQGTVLLSGDPEAFTAHEPTLRALGTVRHVGTD
PGRAAAFDAALLDLFWTTVHGLVHALALARAEGISGTELTPYAQGLPQLLPPLIEDF
ARRADERRHPGDHSSLASAEAGLSHVAHTARARGLDTTVLDAALAVTRRAVEAGH
GSDGVSGLVELFARQATASSTGPATTGPAAAPSNRRRPAGRPR

>yIRED56

MAEKTVSVIGMGSMGSALAKAFLANNHPVTVWNRTASKCTPLEQAGAKVAKSVV
DAAEASQVMVISLLNYEATQSLLQTPEVIEKLKGKVIVQLSTGFPREARESEAWANQ
NGISYLDGAILEYPTGIGKEESVFFYSGSEKVFEENKALLRCLAGNPRFVGKDAGHAA
TLDISTISVYYGASLGFLHGAAICNSEGFPVDKYLSATIPLLSGLIPDTLNMSEKMINE
GSYTAKIATLDVHVAGLKHLLEFIKENRLDTAFMECVLGYCKKAVDMGYTQDELA
AVFEVMKMKVDKKIE

>yIRED57

MAVLGAGSVGSGLVRGLLAGGHPVTVWNRTLRRCEPLSAAGATIVPAPELAIRRAGI
VFSTLSTYAAAREALAQVPDDCLAGRLVVQLSGGTAQEAAVLGDLVRSRGGRYLD
GLLFSYPSAFSDATAILFFAGAEADYEELRRGVFHATSWDSTFVGEHLGTPNQISTMF
EVSIALSMEGFLVGAALAEAVGMSVQTYCDLLVPRFVPTVASMMEESAERVVTGDY
TGEMAIDLWQHVVTRLVGEVESHGVDATALEVVGQQMRRAVAAGFGHGDIAAIYE
AIRRPRDRR

>yIRED58

MSKQAVTIIGLGPMGKAMVNRFLDEGHPVTVWNRTARKADDLVARGAHRAATVE
EALAANEVVVLSLTDFEAMYAILKPAETALAGRVIVNLGSDTPGRAREAAKWATAH
GAGYLTGGVMSAPSGIGAPESFTYYSGQKEIFEAHKETLEVLTGTDYRGSDPGSAAL
YYQLHINMFWTTVISFVHSLAMARANGISAQEFVGPASNSMTDMSAFLEFYAKRIDN
DEHPGDVDRLAMAEASVDHVVHTAKEAGVDHALPAAVLELIRRGMAAGHADDSFT
SLFEVFRKAG

>yIRED59

MGNDEGGRTAVTVVGLGLMGRALAAALLRAGHRTTVWNRTASKADELLAEGALF
APSVHDAVTAAPLVVFCVSDHDALHDLLAPLDGAALDGHTLVNLTSGTPAQARETA
AWAAARGAAYLDGGIMATPQEVGTPAAHVVVCGPRQALEPHRPVLSALAGGTTHL
GEDHGLVALHEMAVMALMWSTLNGFLQGAALLGAAGVDAGAFVPYARQGAEAV
TGWLADHARQIDAGGYPVLDATLGTHLTSMRNLVLESEALGVSTELPRLVAGFADR
AVAGGHGGDSYAALIEEFRNPAPGRPGAPRSEQPEE

>yIRED60

MKPGISVLGTGRMGSALVGAFLKQGYNVAVWNRTKSKCAPLAALGARVATTVRD
AVADAEVVVVNVNDYVTSEALLRQDDVTKGLRGKLIVQLTSGSPRQAREMAAWAR
QHELQYLDGAIMGTPNFIGEPGGTILYSGPGALFEKYKPVLLVLGGNSLHVGSDVGH
ASALDSALLSFLWGSMFGVLQAVSVCEAEGLPLGAYMEYVQATKPMVDGAVTDFV
KRIQTGRFAGDEKTLATVEAHHGALRHLIELCEEHGIHHAVPAAFGQLFQAALQAGH
AQDDFAVLNKFMK
